# Proximity labeling reveals interactions necessary to maintain the distinct apical domains of *Drosophila* photoreceptors

**DOI:** 10.1242/jcs.262223

**Published:** 2024-12-11

**Authors:** Lalitha Sastry, Johnathan Rylee, Simpla Mahato, Andrew C. Zelhof

**Affiliations:** Department of Biology, Indiana University, Bloomington, IN 47405, USA

**Keywords:** Apical, Membrane, Photoreceptor

## Abstract

Specialized membrane and cortical protein regions are common features of cells and are utilized to isolate differential cellular functions. In *Drosophila* photoreceptors, the apical membrane domain is defined by two distinct morphological membranes: the rhabdomere microvilli and the stalk membrane. To define the apical cortical protein complexes, we performed proximity labeling screens utilizing the rhabdomeric-specific protein PIP82 as bait. We found that the PIP82 interactome is enriched in actin-binding and cytoskeleton proteins, as well as proteins for cellular trafficking. Analysis of one target, Bifocal, with PIP82 revealed two independent pathways for localization to the rhabdomeric membrane and an additional mechanism of crosstalk between the protein complexes of the rhabdomeric and stalk membranes. The loss of Bifocal, and enhancement in the *PIP82, bifocal* double mutant, resulted in the additional distribution of Crumbs, an apical stalk membrane protein, to the lateral basal photoreceptor membrane. This phenotype was recapitulated by the knockdown of the catalytic subunit of Protein phosphatase 1, a known interactor with Bifocal. Taken together, these results expand our understanding of the molecular mechanisms underlying the generation of the two distinct photoreceptor apical domains.

## Teaser

**Summary:** The delineation of the *Drosophila* photoreceptor apical membrane into the rhabdomere and stalk is dependent on cross-regulation between two different cortical membrane protein complexes.

## INTRODUCTION

In epithelial cells, the plasma membrane is subdivided into unique apical, basal and lateral regions. The plasma membrane of each region can differ in lipid content and the proteins associated within and in the underlying cortical regions. In the end, this subdivision permits the isolation of distinct cellular functions (Harayama and Riezman, 2018; Riga et al., 2020; Rodriguez-Boulan and Macara, 2014; Schmidt and Grosshans, 2018). In *Drosophila*, the photoreceptors of the adult retina are a prime and well-studied example in understanding the lipid content (Gutorov et al., 2022; Hebbar et al., 2020; Raghu et al., 2012), the mechanisms for protein trafficking to unique plasma membrane regions (Schopf and Huber, 2017; Wagner et al., 2022), and establishment of plasma membrane domains and associated cortical regions (Pichaud, 2018). The *Drosophila* retina consists of hundreds of individual units known as ommatidia. Each ommatidium contains eight photoreceptors specified from subsets of epithelial cells within the eye imaginal discs. Over a period of 96 h, the photoreceptors undergo morphological differentiation, leading to neuronal outgrowth and targeting into the optic lobe. Furthermore, during this process, the key light-sensing organelle, the rhabdomere, is generated.

**Fig. 1.**
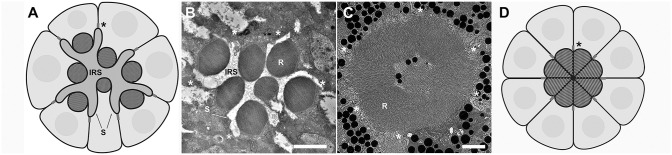
**Comparison of rhabdomere arrangement in open and fused rhabdom systems.** (A) Schematic of an open ommatidium. (B) Transmission electron microscopy (TEM) image of a *Drosophila melanogaster* ommatidium. (C) TEM image of a mosquito, *Culex quinquefasciatus*, ommatidium. (D) Schematic of a fused ommatidium. Asterisks mark the adherens junctions between photoreceptor cells and the boundary of the apical membrane. In the *Culex* photoreceptor, the entire apical surface is dedicated to rhabdomere; in *Drosophila*, the apical membrane is subdivided into the rhabdomere (R) and stalk membranes (S). In an open rhabdom, the rhabdomeres are separated by an extracellular matrix known as the inter-rhabdomeral space (IRS). Scale bars: 2 μm.

The rhabdomere represents a transformation of the apical membrane surface, containing thousands of microvilli critical for housing the phototransduction machinery for the efficient capture and detection of photons. Moreover, the apical domain is defined by adherence junctions between adjacent photoreceptors. In *Drosophila*, the rhabdomere consists of only a portion of the apical membrane and is surrounded and bounded by apical membrane devoid of microvilli, known as the stalk membrane. There are two distinct morphological apical plasma membrane domains (Fig. 1A,B). The stalk membrane has been implicated in protein trafficking to and from the rhabdomere, positioning of each rhabdomere within the ommatidium and secreting the inter-rhabdomeral space (IRS) (Iwanami et al., 2016; Satoh et al., 2005; Schopf and Huber, 2017; Xia and Ready, 2011). As a result, the arrangement of the rhabdomeres within a single ommatidium assumes a derived state known as an open rhabdom (Mahato et al., 2018; Osorio, 2007). Each rhabdomere takes a stereotypical position and is separated from each other within the ommatidium by a secreted extracellular matrix. Combined with the unique wiring of each photoreceptor in the optic lobe, known as neural superposition, this arrangement permits an increase in light sensitivity without a commensurate loss of visual acuity (Agi et al., 2014; Braitenberg, 1967; Kirschfeld, 1967). In the ancestral state, a fused rhabdom, the rhabdomeres of a single ommatidium do not separate from each other and the photoreceptor apical surface does not contain two distinct types of apical membrane (Mahato et al., 2018; Zelhof et al., 2020); the entire apical membrane consists of the rhabdomere (Fig. 1C,D).

The molecular mechanisms that establish and contribute to the evolutionary change from a fused to open rhabdom system remain limited. With respect to the formation of the IRS, the protein Eys, a secreted molecule containing both EGF and Laminin G-like repeats, is critical and represents the extracellular matrix that separates the rhabdomeres (Husain et al., 2006; Zelhof et al., 2006). Moreover, some of the evolutionary mechanisms with respect to the formation of the IRS have been described, including changes in spatial expression of Eys and amino acid differences in Eys protein orthologs (Mahato et al., 2018). Regarding the establishment of the two distinct apical membranes, less is known. Crumbs has been identified as a key molecule for the establishment of the apical stalk membrane. Crumbs is a transmembrane protein containing both EGF and Laminin G-like repeats in its extracellular region, and its intracellular region contains binding sites for both FERM (4.1/Ezrin/Radixin/Moesin) and PDZ (PSD-95/Discs large/ZO-1) domain-containing proteins. Loss-of-function mutations in *crumbs* lead to shortening of the stalk membrane, preventing the full basal extension of rhabdomeres in the retina and resulting in juxtaposed rhabdomeres (Izaddoost et al., 2002; Johnson et al., 2002; Pellikka et al., 2002), whereas overexpression of Crumbs in photoreceptors can lead to an increase in the amount of apical stalk membrane (Pellikka et al., 2002; Pellikka and Tepass, 2017). Conversely, PIP82, a photoreceptor-specific protein unique to open rhabdoms, localizes only to the rhabdomeric membrane, and its spread to the stalk membrane is inhibited by phosphorylation by Atypical protein kinase C (aPKC) (Zelhof et al., 2020). aPKC is localized to the stalk membrane via an interaction with Crumbs (Flores-Benitez and Knust, 2016; Hong et al., 2003; Muschalik and Knust, 2011; Nam and Choi, 2003; Pichaud, 2018; Walther and Pichaud, 2010), thus highlighting a mechanism by which Crumbs can modulate the presence of cortical membrane proteins and maintain a boundary between two apical membrane domains (Zelhof et al., 2020).

Here, we identify cortical protein complexes and explore other potential cellular mechanisms for the delineation of the rhabdomeric and stalk apical membranes in photoreceptors. Utilizing a rhabdomeric-specific cortical protein, PIP82, we identified a protein interactome that was biased towards actin binding-, cytoskeleton- and clathrin-dependent endocytic proteins. Analysis of the actin-binding protein Bifocal (Bahri et al., 1997) demonstrated that, like PIP82, Bifocal localization is limited to only the rhabdomeric portion of the apical domain throughout metamorphosis, and, in tissue culture cells, it localizes with PIP82 to the cell cortical region. However, neither is dependent on the other for localization, but, like PIP82, Bifocal localization expands to the entire apical membrane in the absence of the stalk protein Crumbs. Previous *bifocal* loss-of-function studies demonstrated that *bifocal* was required for proper rhabdomere formation and photoreceptor axonal pathfinding (Babu et al., 2005; Bahri et al., 1997; Helps et al., 2001; Ruan et al., 2002). Here, we demonstrate that loss of Bifocal can redirect the localization of Crumbs to the basolateral photoreceptor membranes and subsequently redirect the secretion of the apical extra cellular matrix, as represented by the incorrect accumulation of Eys in basolateral regions. The loss of both Bifocal and PIP82 compounds the apical organization of the photoreceptors, severely affecting the organization of the rhabdomeres. However, the localization of the alpha subunit of Na^+^ K^+^ ATPase (also known as Atpalpha), a protein that normally localizes to the basolateral membrane, was not affected in the *bifocal, PIP82* double mutant. The exact mechanism of how Bifocal directs the localization of Crumbs is unknown, but we demonstrate that the loss of a Bifocal interactor, the catalytic subunit of Protein phosphatase 1-87B (Pp1-87B), phenocopies the mislocalization of Crumbs. Altogether, our results suggest at least two independent mechanisms for the localization of rhabdomeric cortical membrane proteins and also highlight the cross-regulation between the cortical protein complexes of the rhabdomeric and stalk membranes to ensure proper trafficking, secretion and localization of apical proteins.

## RESULTS

### PIP82 proximity labeling screens

To potentially identify the photoreceptor apical cortical proteins and mechanisms necessary for the proper delineation of the photoreceptor apical membrane into the rhabdomeric and stalk membranes, we took a biochemical approach to identify other proteins associated with or near PIP82. PIP82 was first described as a potential light-dependent phospho-regulated protein (Suri et al., 1998). Subsequent analysis demonstrated that PIP82 is a photoreceptor-specific protein that localizes to the base of the rhabdomeric membrane. PIP82 is a 1195-amino acid protein with a phospho-regulated basic and hydrophobic (PRBH) domain as its only identifiable signature structure (Bailey and Prehoda, 2015; Zelhof et al., 2020); PRBH domains directly interact with phospholipids permitting cortical localization, but, upon phosphorylation by aPKC, the PRBH interaction with the membrane is disrupted (Bailey and Prehoda, 2015). PIP82 is a phosphorylation target of aPKC, and, upon phosphorylation, PIP82 is removed from the cortical membrane. Furthermore, the absence of PIP82 leads to constant misshaping of the rhabdomere because of misdirection of cellular trafficking to the rhabdomere membrane (Zelhof et al., 2020). Our screens were designed to potentially isolate molecules for both the trafficking and retention of PIP82 on a cortical membrane or the rhabdomeric portion of the photoreceptor apical membrane. Instead of direct immunoprecipitation techniques, we chose to utilize an enzyme-catalyzed proximity labeling approach (Kim and Roux, 2016; Roux et al., 2012). Most proximity labeling approaches are based on promiscuous biotin ligase. BioID and BioID2 are produced from *Escherichia coli* BirA and *Aquifex aeolicus* Biotin Ligase, respectively (Kim and Roux, 2016; Roux et al., 2012). Both enzymes require long labeling times, up to 18 h, and high concentrations of exogenous biotin. To circumvent these issues, we used a recently developed iteration called TurboID. TurboID is a very efficient ligase capable of biotinylating nearby proteins very rapidly relative to previous BirA derivatives and without the addition of exogenous biotin (Branon et al., 2018). We elected to utilize this form of BioID given its theoretically greater chance of detecting both stable and dynamic interactions.

**Fig. 2.**
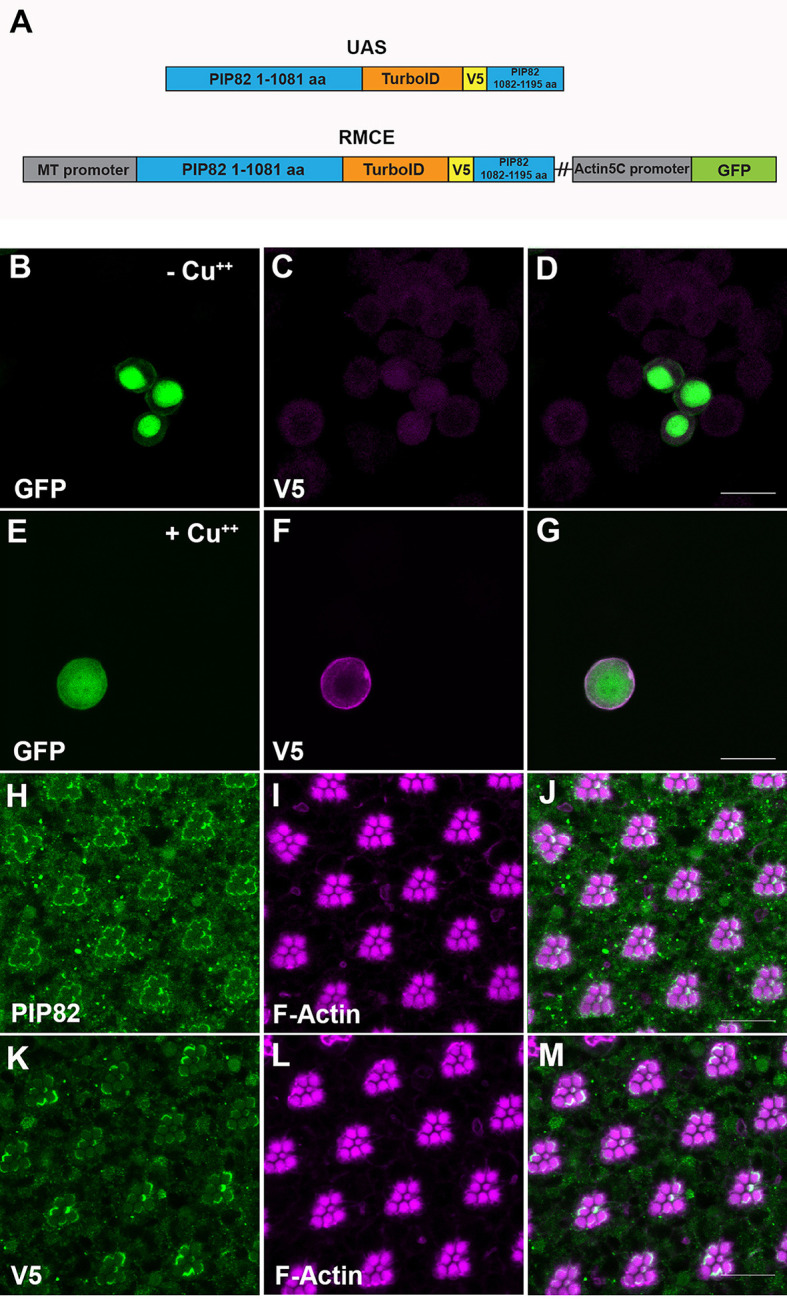
**PIP82-TurboID construct and expression in *Drosophila* tissue culture cells and photoreceptor cells.** (A) Schematic of PIP82-TurboID construct utilized for expression in photoreceptors (UAS cassette) and *Drosophila* S2 cells [recombination-mediated cassette exchange (RMCE) cassette]. In the RMCE cassette, PIP82-TurboID is under the control of the inducible *Drosophila metallothionein* (MT) promoter and linked with GFP expression under the control of the *Actin 5C* promoter. aa, amino acids. (B–D) Immunostaining against the V5 epitope (magenta) of S2 cells transfected with the PIP82-TurboID RMCE cassette plasmid in the absence of copper (Cu^++^), GFP marks the transfected cells. (E–G) Immunofluorescence staining against the V5 epitope (magenta) of S2 cells transfected with the PIP82-TurboID cassette plasmid in the presence of copper (Cu^++^). (H-J) Immunostaining of PIP82 (green) and F-Actin (magenta) in wild-type adult photoreceptors. (K–M) Immunostaining of V5 epitope (green) and F-actin (magenta) in *Pph13-Gal4*; *UAS-PIP82-TurboID* transgenic adult photoreceptors. Scale bars: 10 μm. Native PIP82 is 132 kDa, and the addition of the V5 epitope tag and TurboID adds an additional 35 kDa.

One screen was to express our PIP82-TurboID protein during the time of endogenous expression of PIP82 in differentiating photoreceptors. Subsequently, protein lysates from heads from newly emerged animals (<24 h upon eclosion) were generated for analysis. We chose not to dissect retinas owing to the difficulty of isolating suitable number of retinas coupled with processing of the samples. However, utilizing the entire head could result in a high background, and specific hits might be difficult to identify. Therefore, we complemented our *in vivo* analysis with a screen in *Drosophila* S2 tissue culture cells. PIP82 is not expressed in S2 cells, but transient transfections with PIP82 in S2 cells demonstrated that PIP82 adopts a cortical membrane localization pattern that is disrupted by aPKC phosphorylation, similar to PIP82 localization in photoreceptors (Zelhof et al., 2020). As such, general factors for PIP82 trafficking and retention on the cortical membrane could be identified. For both screens, TurboID was fused within the carboxy terminus of PIP82, between amino acids 1081 and 1082 (Fig. 2A).

To limit expression in photoreceptors, the fusion protein was placed under control of UAS (Brand and Perrimon, 1993) and expressed only in differentiating photoreceptors with the use of Pph13-GAL4 (Liang et al., 2016; Rylee et al., 2022); PIP82 is a direct transcriptional target of Pph13 (Liang et al., 2016). For experiments in tissue culture cells, we believed that transient transfections would not result in a large enough homogeneous population of cells expressing PIP82-TurboID. As such, we created a stable cell line for the expression of PIP82-TurboID. To generate this stable cell line, we utilized the attP/PhiC31 integrase system and recombination-mediated cassette exchange (RMCE) to insert a copy in which the PIP82-TurboID fusion protein is under the inducible *Drosophila metallothionein* (also known as *MtnA*) promoter into the attP site in the S2R+ 99F8 *Drosophila* cell line (Mariyappa et al., 2021). In photoreceptors and S2 cells, western blot analysis confirmed the expression of PIP82-TurboID (Fig. S1). Additionally, immunofluorescence analysis of the fusion protein confirmed the identical localization pattern as the untagged protein in S2 cells (Fig. 2B–G) and in photoreceptor cells (Fig. 2H–M). The additional expression of PIP82-TurboID did not result in any gross abnormalities of the photoreceptor morphology, as assayed by F-Actin immunofluorescence (Fig. 2K–M). We detected enrichment of biotinylated proteins upon the expression of PIP82-TurboID in tissue culture cells and, to a lesser extent, in photoreceptors (Fig. S2).

For our analysis of the PIP82 interactome in photoreceptors, four replicates, each consisting of 100 heads, were generated from PIP82-TurboID flies and *w^1118^* flies. Utilizing Significance Analysis of INTeractome (SAINT) (Choi et al., 2011), the analysis did not highlight potential specific versus non-specific interactions. However, for comparison to our S2 experiments, we sorted the data based upon the following criteria: we identified proteins that were enriched upon expression of PIP82-Turbo-ID versus the *w^1118^* control either exclusively or by at least threefold. This analysis resulted in 13 protein interactors (Table S1). For characterizing the S2 cell PIP82 interactome, we examined three replicates of 1×10^8^ cells of S2 PIP82-TurboID compared to the same number of replicates and cell totals of S2 cells. In contrast to what was found for photoreceptor cells, SAINT analysis identified 58 interactions above the cutoff of ≥0.8 (Fig. S4 and Table S2). PAthway, Network and Gene-set Enrichment Analysis (PANGEA) (Hu et al., 2023) demonstrated that this set of interactors was enriched for actin binding, cytoskeleton proteins and proteins involved in synaptic plasticity (Fig. S3). Ten of the 58 interactors have been implicated in *Drosophila* retina development (Fig. S4). Comparing the datasets from S2 cells with those from *Drosophila* adult heads, we found only two genes that overlapped between the datasets, *Supervillin* and *bifocal*. Supervillin is implicated in regulating the actin cytoskeleton (Crowley et al., 2009), and *bifocal* is required for proper photoreceptor differentiation (Babu et al., 2005; Bahri et al., 1997; Helps et al., 2001; Ruan et al., 2002), including rhabdomere formation.

### Generation of *bifocal* mutants and antibody

The initial *bifocal* mutant was isolated in a P-element transposon mobilization screen in the 10D cytogenic region. Subsequent characterization of null alleles demonstrated that *bifocal* was required for proper rhabdomere formation and photoreceptor axonal pathfinding (Babu et al., 2005; Bahri et al., 1997; Helps et al., 2001; Ruan et al., 2002). Bifocal is also capable of binding both F-Actin and microtubules (Sisson et al., 2000). Like *PIP82* mutants, *bifocal* mutants did not affect photoreceptor specification but rather photoreceptor differentiation. *bifocal* mutant rhabdomeres are mishappen and often have a split duplicated phenotype (Bahri et al., 1997). However, a potential mechanism for the *bifocal* phenotype or a role in delineating or maintaining the apical photoreceptor membrane was not described. To further characterize the role of Bifocal in apical membrane biogenesis, we needed to recreate *bifocal* null mutant alleles and generate an antibody against Bifocal. To generate a *bifocal* null allele, the 240 bp second exon was deleted and replaced with a floxed cassette containing the 3XP3 promoter driving DsRed expression via CRISPR/Cas9 homologous recombination (Fig. S5). Subsequently, the cassette was removed to generate *bifocal^exon2del^* mutant allele, resulting in a truncation of the protein to 127 amino acids, containing the first 104 endogenous amino acids and 23 additional amino acids. Our antibody confirmed the localization of Bifocal on the apical membrane (Fig. S6A–C). Immunofluorescence was lost in our mutant allele, and disruption of F-Actin localization was observed as previously described (Fig. S6D–F). Transmission electron microscopy (TEM) demonstrated that the *bifocal^exon2del^* mutant mimics the previously described rhabdomere defect (Fig. S6G,H).

### Bifocal and PIP82 interactions

**Fig. 3.**
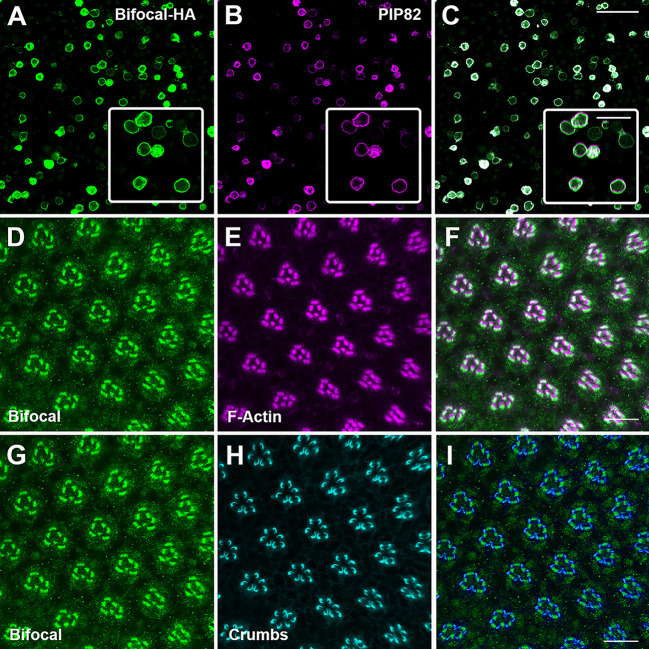
**Bifocal and PIP82 spatial expression in S2 cells and differentiating photoreceptors.** (A–C) Immunostaining of S2 cells co-transfected with Bifocal-HA (green) and PIP82 (magenta). Inset shows a higher-magnification view. Scale bars: 50 μm and 10 μm (inset). (D–I) Immunostaining of photoreceptors of wild-type retina at 72 h after puparium formation (APF) with Bifocal, F-Actin and Crumbs. (D–F) Comparison of Bifocal (green) and F-Actin (magenta). (G–I) Comparison of Bifocal (green) and Crumbs (cyan). Scale bars: 10 μm.

Given the close proximity and similar spatial and temporal expression patterns in photoreceptors, we examined whether there is a functional relationship between PIP82 and Bifocal. We first examined colocalization in S2 cells and photoreceptor cells. Based on our interactome data and confirmed by modENCODE data (Mod et al., 2010), *bifocal* is expressed in *Drosophila* S2 cells. Given that both antibodies that recognize PIP82 and Bifocal were raised in rabbits, we generated a hemagglutinin (HA) epitope tag version of Bifocal. Upon co-transfection of PIP82 and Bifocal-HA, we observed colocalization of PIP82 and Bifocal on the cortical membrane of S2 cells (Fig. 3A–C). In photoreceptors, we know that PIP82 accumulates on the apical photoreceptor cell surface around 48 h and, by 72 h, PIP82 localizes to the base of the rhabdomere; its boundary of expression is framed by Crumbs localization on the stalk membrane on either side of the rhabdomere (Zelhof et al., 2020). Therefore, we expected to see the same localization pattern for Bifocal. Endogenous Bifocal accumulated on the apical surface of photoreceptor cells at 48 h after puparium formation (APF) (Fig. S6A–C), and, by 72 h APF, Bifocal had the identical localization pattern as reported for PIP82; Bifocal localized to the base of the rhabdomere and its boundary was framed by Crumbs localization on the apical stalk membrane (Fig. 3D–I).

**Fig. 4.**
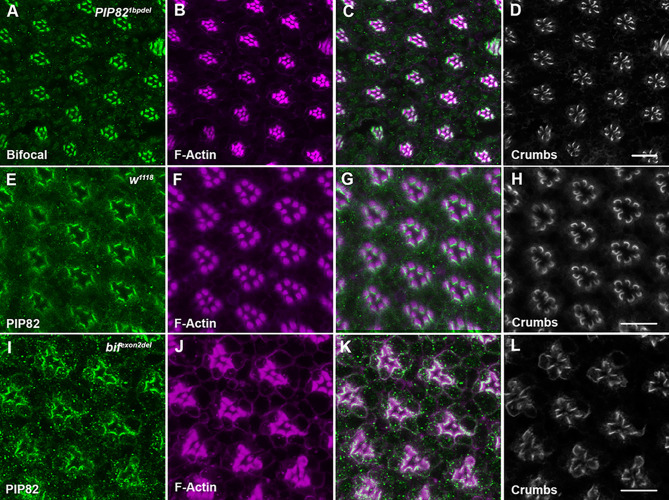
**Neither Bifocal nor PIP82 is dependent on the other for localization on the photoreceptor apical surface.** (A–C) Immunostaining of Bifocal (green) and F-Actin (magenta) in a PIP82 mutant (*PIP82^1bpdel^*) retina at 72 h APF. (D) Crumbs localization in the same ommatidia as A–C. (E–G) Immunostaining of PIP82 (green) and F-Actin (magenta) in a wild-type retina (*w^1118^*). (H) Crumbs localization in the same ommatidia as E–G. (I–K) Immunostaining of PIP82 (green) and F-Actin (magenta) in a *bifocal* mutant (*bifocal^exon2del^*) retina. (L) Crumbs localization in the same ommatidia as I–K. Scale bars: 10 μm.

To determine whether either PIP82 or Bifocal is dependent on the other for proper localization in photoreceptors, we examined Bifocal localization in *PIP82* mutants and PIP82 localization in *bifocal* mutants (Fig. 4). In both cases, PIP82 and Bifocal could localize to the apical surface in the absence of the other protein. Given that neither PIP82 nor Bifocal is dependent upon the other for localization suggests the possibility of independent methods to localize to the rhabdomeric cortical region of the membrane. PIP82 localization is dependent upon its PRBH domain and Bifocal localization through its interaction with Actin or microtubules (Sisson et al., 2000). We previously demonstrated that PIP82 localization to the rhabdomeric cortical region was dependent upon Crumbs via localization and subsequent phosphorylation by aPKC (Zelhof et al., 2020). Like PIP82, Bifocal localization was also altered in *crumbs* mutant photoreceptors, in which Bifocal was localized to the entire apical surface (Fig. S7A–C), reinforcing the idea that Crumbs is a key factor for delineating the cortical boundary between the rhabdomeric and stalk membrane. Nonetheless, there was not a global defect in cellular trafficking upon the loss of Crumbs. The alpha subunit of the Na^+^ K^+^ ATPase localized correctly to the photoreceptor basolateral membranes in *crumbs* mutant cells (Fig. S7D–F).

### Bifocal, the mislocalization of Crumbs and crosstalk between apical membranes

**Fig. 5.**
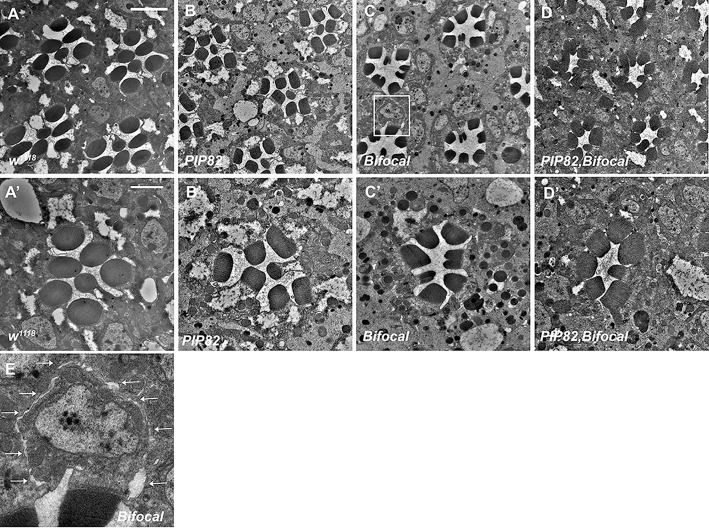
**The combinatorial loss of PIP82 and Bifocal reshapes the arrangement of the rhabdomeres within an ommatidium.** (A,A′) TEM image of a wild-type retina. Note the round shape, separation and trapezoidal arrangement of the rhabdomeres within each ommatidium. (B,B′) TEM image of a *PIP82* mutant retina. Note the squared elongated shape, but separation and trapezoidal arrangement of the rhabdomeres within each ommatidium is maintained. (C,C′) TEM image of a *bifocal* mutant retina. Note the split shape and separated rhabdomeres of different photoreceptors within each ommatidium. The loss of the trapezoidal arrangement in some ommatidia is noticeable. (D,D′) TEM image of a *PIP82, bifocal* double mutant retina. Note the loss of the split shape of the rhabdomeres. The rhabdomeres are close to or touching each other and have lost their trapezoidal arrangement within an ommatidium. (E) Enlarged section of the boxed region in C. The arrows denote extracellular space around the basolateral region of photoreceptor. Scale bars: 5 μm (A) and 2 μm (A′).

**Fig. 6.**
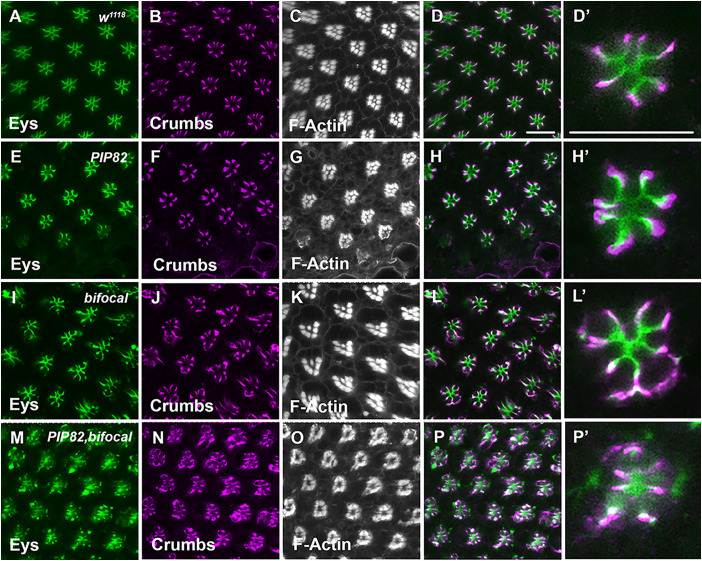
**Mislocalization of Crumbs and Eys in the *PIP82, bifocal* double mutant.** Immunostaining of Eys (green), Crumbs (magenta) and F-Actin (white) in wild-type and mutant retinas at 84 h APF. (A–D) Wild-type retina. Panels D and D′ represent a merged image of panels A and B. (E–H) *PIP82* mutant retina. Panels H and H′ represent a merged image of panels E and F. (I–L) *bifocal* mutant retina. Panels L and L′ represent a merged image of panels I and J. (M–P) *PIP82, bifocal* double mutant retina. Panels P and P′ represent a merged image of panels M and N. Scale bars: 10 μm.

To examine the functional consequence of photoreceptors lacking *bifocal* and *PIP82*, we generated a double mutant. The double mutant was viable and fertile. Utilizing TEM, we noted two distinct phenotypes in the double mutant. The first was that the trapezoid arrangement of the rhabdomeres was lost and instead the rhabdomeres assumed a more circular arrangement compared to that of wild type or individual *PIP82* and *bifocal* mutants (Fig. 5). In addition, the rhabdomeres were not completely separated from each other and contacted each other (Fig. 5D). The observed phenotype was reminiscent of the loss of *crumbs*. In mutant *crumbs* photoreceptor cells, the stalk membrane is not fully elaborated and the lateral sides of distinct rhabdomeres of each individual ommatidium often contact each other (Bulgakova and Knust, 2009; Izaddoost et al., 2002; Muschalik and Knust, 2011; Pellikka et al., 2002; Pellikka and Tepass, 2017; Richard et al., 2009). Previous data demonstrated that the loss of PIP82 does not change Crumbs localization to the apical stalk membrane, nor does it affect the secretion of Eys to generate the IRS (Fig. 6E–H) as compared to a wild-type retina (Fig. 6A–D), but Crumbs is essential for limiting PIP82 localization to the base of the rhabdomere on the apical surface (Zelhof et al., 2020). Based on the *bifocal* mutant TEM, we expected to observe a phenotype like *PIP82* with respect to Crumbs and Eys localization. However, we observed the extension of Crumbs and Eys localization along the basolateral membrane in photoreceptors and, in some cases, surrounding an entire photoreceptor cell; the mislocalization of Crumbs correlates with the aberrant secretion of Eys (Fig. 6I–L). Upon closer examination of our *bifocal* TEM images, we could identify the manifestation of the mislocalization of Crumbs and Eys phenotype. We detected an extracellular space extending around the entire photoreceptor (Fig. 5C,E). Based on immunofluorescence data, the adherence junctions still formed correctly in the double mutant, as indicated by the proper accumulation of Armadillo at the apical junctions between photoreceptors (Fig. S8A–C), and localization of the alpha subunit of the Na^+^ K^+^ ATPase to the basolateral membranes was not affected (Fig. S8D–F). In *PIP82, bifocal* double mutant, we observed complete disruption of the proper distribution and accumulation of Eys and Crumbs. Eys has limited accumulation in the IRS and can be detected in the cell cytoplasm (Fig. 6M,P). The combination of the loss of Crumbs, demarcating the stalk membrane (Fig. 6N,P), and the failure to secrete Eys corresponds with the very limited separation of the rhabdomeres and the loss of the trapezoidal arrangement of the rhabdomeres within each ommatidium (Fig. 6O). Our data indicate that, in the absence of both PIP82 and Bifocal, the initial establishment of the apical membrane is normal. We observed the generation and presence of adherence junctions and localization of Crumbs to the stalk membrane. However, the loss of Bifocal permitted accumulation of Crumbs to other membranes and subsequent mislocalization of Eys. As such, the mislocalization of Crumbs and the corresponding aberrant secretion of Eys support the idea that PIP82 and Bifocal are necessary to maintain the correct trafficking and localization of both rhabdomeric and stalk membrane components.

### Loss of Protein phosphatase 1 catalytic subunit phenocopies the mislocalization of Crumbs

**Fig. 7.**
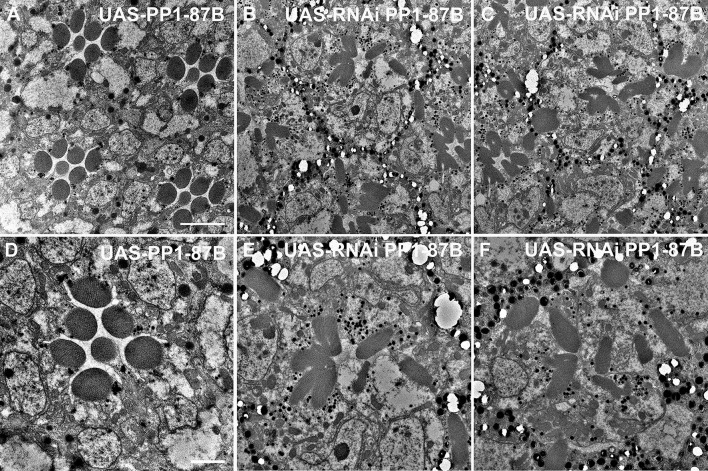
**TEM analysis of loss and gain of function of Pp1-87B in photoreceptors.** (A–C) Rhabdomere structure and organization in gain-of-function Pp1-87B photoreceptors (A; Pph13-GAL4; UAS-Pp1-87B) compared to loss-of-function Pp1-87B ommatidia (B,C; Pph13-GAL4, UAS-RNAi Pp1-87B). Scale bar: 5 μm. (D–F) Higher-magnification images of rhabdomere structure and organization in gain-of-function Pp1-87B photoreceptors (D; Pph13-GAL4; UAS-Pp1-87B) compared to loss-of-function Pp1-87B ommatidia (E,F; Pph13-GAL4, UAS-RNAi Pp1-87B). Scale bar: 2 μm.

**Fig. 8.**
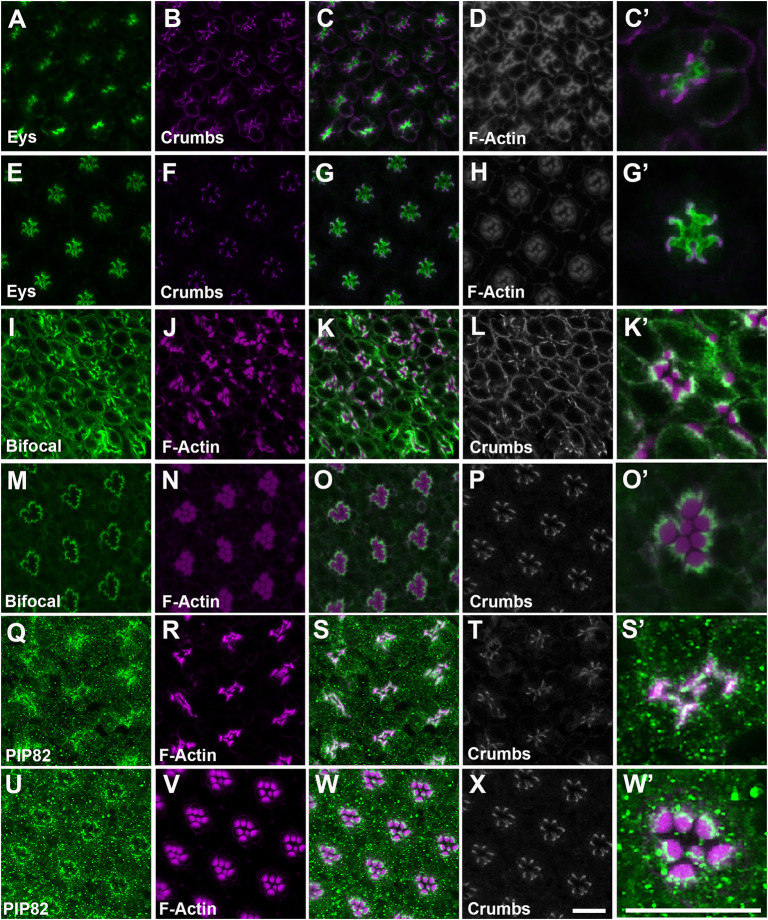
**The loss of Pp1-87B function in photoreceptors mimics the mislocalization of Crumbs in *bifocal* mutants.** (A–X) Immunostaining of loss-of-function (A–D,I–L,Q–T) and gain-of-function (E–H,M–P,U–X) Pp1-87B retinas at 84 h APF (A–P) and 72 h APF (Q–X). (A–H) Comparison of Eys (green), Crumbs (magenta) and F-Actin (gray) localization. Panels C and C′ represent merged images of panels A and B; panels G and G′ represent merged images of E and F. (I–P) Comparison of Bifocal (green), F-Actin (magenta) and Crumbs (gray) localization. Panels K and K′ represent merged images of panels I and J; panels O and O′ represent merged images of M and N. (Q–X) Comparison of PIP82 (green) F-Actin (magenta) and Crumbs (gray) localization. Panels S and S′ represent merged images of panels Q and R; panels W and W′ represent merged images of U and V. Scale bars: 10 μm.

Whereas the data presented here link Bifocal with PIP82 at the rhabdomeric apical cortical membrane, and their localization is independent of each other, previous characterization of *bifocal* mutants has identified a few interactors, despite the lack of any identifiable protein domains (Bahri et al., 1997; Helps et al., 2001; Ruan et al., 2002). The catalytic subunit of Pp1-87B was identified as a Bifocal-binding protein in a yeast two-hybrid screen, and expression of a cDNA in which the Bifocal Pp1-87B-binding site is mutated does not rescue *bifocal* mutant phenotypes (Helps et al., 2001). In addition, *bifocal* and *Pp1-87B* genetically interact to control the targeting of photoreceptor axons into the brain (Babu et al., 2005). However, *Pp1-87B* null mutants have a defect in mitosis and are larval lethal (Axton et al., 1990; Dombrádi et al., 1990), and thus the role of Pp1-87B in rhabdomere morphology and organization has not been explored. To test whether the phenotypes observed upon the loss of Bifocal are related to an interaction with PP1, we utilized RNA interference (RNAi) to characterize Pp1-87B loss-of-function phenotypes as well as overexpression of Pp1-87B in photoreceptor cells (Moreira et al., 2019; Song et al., 2023). We utilized Pph13-GAL4 to express either Pp1-87B RNAi or UAS-Pp1-87B and to avoid any earlier development requirements of Pp1-87B in eye development and cell-fate specification (Liang et al., 2016; Rylee et al., 2022). First, we found that the overexpression of Pp1-87B did not result in any detectable morphological defects (Fig. 7A,D) or mislocalization of apical cortical proteins (Fig. 8) and thus served as a control for comparisons to the Pp1-87B RNAi loss-of-function phenotypes. Conversely, the knockdown of Pp1-87B in photoreceptors resulted in complete disorganization of the morphology and placement of rhabdomeres; the formation of rhabdomeres was observed on any of the membrane surfaces of the photoreceptor and, in many cases, there was elimination of the IRS (Fig. 7B,C,E,F). Strikingly, even though the Pp1-87B loss-of-function phenotype appears more severe than that of the *bifocal* mutant and the *PIP82, bifocal* double mutant, we observed the identical mislocalization of Crumbs and Eys to the basolateral surfaces, as observed with the loss of Bifocal, suggesting that the incorrect localization of Crumbs in a *bifocal* mutant is through the activity of Pp1-87B (Fig. 8A–G). In addition, the loss of Pp1-87B or overexpression of Pp1-87B did not affect the enrichment of Bifocal (Fig. 8I–P) or PIP82 (Fig. 8Q–X) at the rhabdomeric membranes, even when the rhabdomeres formed anywhere in the photoreceptor. Overall, it appears that Pp1-87B activity is critical for properly defining the apical membrane surface for both the proper placement and formation of the rhabdomeres, and the mislocalization of Crumbs in a *bifocal* mutant correlates with the loss of Pp1-87B activity, presumably at the apical cortical region.

## DISCUSSION

### PIP82 protein interactome

To further identify molecular mechanisms responsible for delineation of the photoreceptor apical membrane into the rhabdomeric and stalk membrane, we screened for interactors of PIP82. PIP82 was chosen because its localization defines a molecular mechanism for the isolation of proteins to a portion of the photoreceptor apical membrane domain. PIP82 only localizes to the apical region beneath the rhabdomeric microvilli via its intrinsic PRBH domain (Bailey and Prehoda, 2015; Zelhof et al., 2020). The PRBH domain directly binds phospholipids (Bailey and Prehoda, 2015). PIP82 is actively excluded from the stalk membrane via phosphorylation of the PRBH domain by aPKC anchored and localized to the stalk membrane by Crumbs (Nam and Choi, 2003; Nunes de Almeida et al., 2019; Walther and Pichaud, 2010). In *PIP82* mutants, the delineation of both the stalk membrane and rhabdomere is still present; there is no change in Crumbs localization but the rhabdomere itself is not maintained during adult life (Zelhof et al., 2020). As such, PIP82 does not directly specify either membrane but, more importantly, revealed a molecular mechanism for the restriction or delivery of cortical proteins to a particular region of the membrane. We believed that investigating the potential trafficking and retention of PIP82 on the rhabdomeric membrane would reveal other key regulatory processes for the generation and maintenance of the apical membrane domains.

Our PIP82 interactive study was designed to detect transient and stable interactions with the use of TurboID (Branon et al., 2018). Our screen was enhanced using *Drosophila* tissue culture cells and the utility of a stable cell line in which the cortical localization of PIP82 was reproduced to complement the *in vivo* screen with photoreceptors. However, with respect to the photoreceptor interactome, we chose to utilize and analyze whole-head extracts, but our signal-to noise ratio could not provide enough confidence to isolate true interactors from false interactors based on SAINT analysis (Choi et al., 2011). Recently, biotin proximity labeling was reported for rhodopsin (Rh1; also known as NinaE) in *Drosophila* (Feizy et al., 2024). In contrast to our screen, the screened lysates were first analyzed by gel electrophoresis for specificity and only differential bands were subject to liquid chromatography with tandem mass spectrometry (LC-MS/MS) analysis. Interestingly, PIP82 was identified as an interactor, and our previous results had demonstrated that Rh1 is misdirected in *PIP82* mutants (Zelhof et al., 2020). As such, our S2 interactome did recover several proteins involved with the actin cortical network and in protein trafficking, in particular endocytosis. With respect to endocytosis, we recovered Liquid facets and Liquid facets related, two Epsin proteins, Epsin15 (Eps-15) and Auxilin. Interestingly, the functions of these proteins have been investigated with respect to *Drosophila* eye development and specification (Eun et al., 2007; Hamanaka and Meinertzhagen, 2010; Kandachar et al., 2008; Majumdar et al., 2006; Overstreet et al., 2004), but their functions later in photoreceptor differentiation have not been described. Combined with the previous characterization of *PIP82* null phenotypes, our results suggest that PIP82 is critical for maintaining the rhabdomeric membrane by interacting with the actin cortical network and trafficking proteins to direct material or maintain the complexes on the apical cortical membrane. Subsequent studies will be needed to further define how PIP82 interacts with other potential candidates.

### Bifocal – an old but new player

Both our datasets identified Bifocal as a PIP82 interactor. Bifocal was previously identified a critical factor in photoreceptor differentiation, affecting both rhabdomere morphogenesis and photoreceptor axonal targeting (Babu et al., 2005; Bahri et al., 1997; Helps et al., 2001; Ruan et al., 2002). With the regeneration of a null allele for *bifocal* and antibodies raised against Bifocal, we examined the interaction with PIP82 and re-examined the role of Bifocal at the apical photoreceptor membrane. Both Bifocal and PIP82 localize to the cortical membrane in *Drosophila* tissue culture cells and share the same spatial and temporal expression profile in photoreceptors. However, our analysis of mutants demonstrated that neither is dependent upon the other for localization. Both localize only to the rhabdomeric cortical portion of the membrane flanked by Crumbs. The lack of interdependency for localization might be due to the ability of PIP82 to directly bind phospholipids (Bailey and Prehoda, 2015) and to Bifocal binding actin and microtubules (Sisson et al., 2000). But our analyses confirmed that both are in proximity to each other, contributing to the uniqueness of the rhabdomeric apical region, and the disruption of the rhabdomere structure in a *bifocal* mutant is thought to manifest via the interaction of Bifocal with actin (Bahri et al., 1997). Our analysis revealed that Crumbs is mislocalized in a *bifocal* mutant. In addition to the normal localization of Crumbs to the stalk membrane, Crumbs can be found on the basolateral surfaces; there were no obvious defects in the stalk membrane. Crumbs has multiple roles in photoreceptor differentiation, including photoreceptor cell fate choice (Pojer et al., 2021) to establishing and maintaining photoreceptor apical-basal polarity. With respect to polarity, Crumbs not only helps in delineating the apical membrane from the basolateral membrane (Bulgakova and Knust, 2009; Pichaud, 2018) but also organizes the phospholipid homeostasis of the apical membrane and is necessary for efficient apical secretion (Lattner et al., 2019). As such, the disruption in rhabdomere morphogenesis observed in *bifocal* mutants might be directly related to the disruption of trafficking to the apical surface and not necessarily due to Bifocal directing or regulating actin dynamics. Such a model is supported by the inappropriate secretion of the extracellular matrix protein Eys to the basolateral surface of the photoreceptors in *bifocal* mutant photoreceptors; the sites of secretion correlate with the aberrant localization sites of Crumbs.

### Phosphorylation states and apical membrane domains

The connection between the additional localization of Crumbs to the basolateral membranes and Bifocal function is unclear. The correct recruitment of Crumbs to the apical surface is dependent upon proteins associated with the Par complex – Par-6, aPKC and Bazooka – and Cdc42 drives apical identity (Nunes de Almeida et al., 2019). Our data reveal that PIP82 and Bifocal are early indicators of the rhabdomeric membrane and that subsequent delineation between the stalk and rhabdomere membrane is dependent on Crumbs. In the case of PIP82, our previous study determined that the direct phosphorylation of PIP82 by the Crumbs-aPKC complex limits PIP82 localization (Zelhof et al., 2020). Here, we observed the same phenotype for Bifocal upon the removal of Crumbs; Bifocal was then located along the entire apical surface in the absence of Crumbs. However, additional studies will be needed to determine whether the same mechanism of phosphorylation by the aPKC is also delimiting Bifocal localization to the rhabdomeric membrane.

In addition, our results have revealed an interaction between the proteins of the rhabdomeric membrane and the proteins of the stalk membrane to maintain the identity of both apical membranes. The stalk membrane is defined by Crumbs localization. In the absence of Bifocal, Crumbs localization is no longer limited to the apical surface even though initial apical-basal polarity appears to be established by the formation of adherens junctions, the formation of rhabdomeres only on the putative apical surface and apical secretion of Eys. Here, we demonstrated that the known interaction between Bifocal and the catalytic subunit of Pp1-87B could explain the change in Crumbs localization. The loss of Pp1-87B in photoreceptors phenocopies the mislocalization of Crumbs. Whereas aPKC is a serine/threonine kinase and Pp1-87B is a serine/threonine phosphatase, suggesting that the balance of phosphorylation states within the photoreceptor is critical for maintaining Crumbs on the apical surface. Crumbs is phosphorylated by aPKC, and this phosphorylation is required for proper localization in epithelial cells (Sotillos et al., 2004). However, how phosphorylation states of Crumbs and the associated Par complex (aPKC, Par-6 and Bazooka) change upon defining and maintaining the apical surface and the delineation of the stalk membrane have not been described, nor have the potential targets of PP1-87 been identified in *Drosophila* photoreceptor cells.

Lastly, even though the loss of Pp1-87B phenocopies the mislocalization of Crumbs, the resulting phenotypes we observed were not equivalent to a *bifocal* mutant. The Pp1-87B loss-of-function phenotypes observed align more with a complete loss of a defined apical surface and apical-basal polarity. Rhabdomeres, the signature structure of the apical surface, form on any membrane surface, and the secretion and formation of a defined IRS is severely limited. The phenotype is reminiscent of the defined role of Pp1-87B in dividing *Drosophila* epithelial cells. Pp1-87B is required to counteract the activity of the apical aPKC-Crumbs complex by re-establishing the cortical localization of Lethal giant larvae [Lgl; also known as L(2)gl] in the basolateral region after cell division; the dephosphorylation of Lgl by Pp1-87B restores Lgl basolateral cortical membrane localization required to maintain apical basal polarity (Moreira et al., 2019). Future studies will be able to test similar interactions in photoreceptors.

### Bifocal, PIP82 and Crumbs, and the transition from fused to open rhabdoms

The transition from fused to open rhabdoms is marked by the creation of an extracellular matrix that separates each rhabdomere and the division of the apical membrane into the stalk and rhabdomere portions. We know that PIP82 originated *de novo* in the lineage leading to brachyceran Diptera, which is characterized by the transition from fused to open rhabdoms, and localization is influenced by aPKC and Crumbs (Zelhof et al., 2020). Here, we demonstrate that PIP82 and Bifocal are both required for proper morphogenesis but not required for the formation of the rhabdomeres. This can also be said for Crumbs; Crumbs function is not required for rhabdomere formation (Izaddoost et al., 2002; Pellikka et al., 2002), but our data suggest that it might directly or indirectly aid in the correct secretion of Eys to form the IRS and be necessary for stalk membrane formation. Therefore, as observed with our results, the *PIP82, bifocal* double mutant phenotype still permits the formation of rhabdomeres, but the subsequent mislocalization of Crumbs and the inefficient and misdirection of Eys secretion leads to a rhabdomere organization that resembles a fused rhabdom. The rhabdomeres’ stereotypical position within each rhabdom is not maintained; the rhabdomeres are closely juxtaposed and no longer positioned correctly within each ommatidium for proper vision. Based on our data, we would speculate that the function of Crumbs would not be required in fused rhabdoms; the stalk membrane is not present, and Eys is not expressed in photoreceptors and thus not needed for apical secretion (Mahato et al., 2018; Zelhof et al., 2006). Moreover, our data indicate that PP1 activity is critical for establishing apical basal polarity and would still be required in a fused system to establish the identity of the apical membrane.

## MATERIALS AND METHODS

### PUASTattB PIP82-TuboID fusion construct

TurboID was amplified from TurboID-V5_pRS415 (Addgene #107167) (Branon et al., 2018). PIP82 coding sequence was amplified as two fragments to insert TurboID between PIP82 amino acids 1081 and 1082. PIP82-TurboID was assembled as three PCR fragments using NEBuilder HiFi mix (NEB) into Xho1 linearized pUASTattB (RRID: DGRC1419) (Bischof et al., 2007). Whole-plasmid sequencing (Primordium Labs) was completed to confirm construct. The resulting construct was injected into fly embryos containing a second chromosome attP landing site (attP40w) by Rainbow Transgenic Flies Inc. (Camarillo, CA). The embryos were reared and crossed to *w^1118^* flies, and the resulting progeny were screened for incorporation of the mini-white transgenic marker.

### RMCE-MT-PIP82-TurboID construct

pUASTattB-PIP82-tID was linearized using NotI. Methallothionine (MT) promoter was amplified from pMT-EGFP-Actin5c (RRID: DGRC1461) plasmid and cloned into pUASTattB-PIP82-TurboID using NEBuilder HiFi mix (NEB). Primers used for amplifying MT promoter were as follows: MT PIP82 FOR, 5′-ATTGGGAATTCGTTAACAGATCTGCCGTTGCAGGACAGGATGTGGT-3′; MT PIP82 REV, 5′-CGGGGGATCCACTAGTTCTAGAGCCGCCTTTAGTTGCACTGAGATG-3′.

MT::PIP82-tID was inserted into pUC57-Act5c::GFP (RRID: DGRC1545) by amplifying MT::PIP82-TurboID from pUASTattB-PIP82-tID, and pUC57-act57::GFP was linearized with NotI. The two fragments were assembled using NEBuilder HiFi mix (NEB). Whole-plasmid sequencing (Primordium Labs) was completed to confirm construct. Primers used to amplify MT::PIP82-tID were as follows: MTPIP82 to ACT5C FOR, 5′-ATTCGGTACCGAGCTCAAGCTTGCCGTTGCAGGACAGGATGTGGTGC-3′; MTPIP82 to ACT5C REV, 5′-CTGGCATAATTGTATGAGGCCTGCGTCGACACTAGTGGATCCAGACATG-3′.

### PIP82-TurboID stable transgenic cell line

The attP/PhiC31 integrase system and RMCE was utilized to generate stably integrated transgenic cell lines that contain copies of the transgene at the desired locus in *Drosophila* cells (Mariyappa et al., 2021). S2R+ 99F8 cells were grown in M3 medium (Sigma) containing bactopeptone, yeast extract and supplemented with 10% fetal bovine serum. For RMCE transfections, 2 ml (1.5×10^6^/ml) S2R+ 99F8 cells were plated in a six-well plate and were transfected with 500 ng MT PIP82-TurboID -Act5c GFP and 500 ng Act::phiC31 integrase (RRID: DGRC 1368), using Effectene Transfection reagent (QIAGEN) as per the manufacturer's instructions. Transfected cells were cultured for at least 10 days to amplify the cultures to 2× 10 cm dishes before the bulk sorting of the cells. Fluorescent-activated cell sorting was performed at least 10 days post-transfection on a FACS Aria II [Flow Cytometry Core Facility, Indiana University (IU), Bloomington, IN]. Live cells were sorted by exclusion of DAPI, detected by excitation with a 407 nm laser. To detect EGFP or dsRED positivity, a 488 nm or a 561 nm laser was used, respectively. Cells at a density of 5-10 million/ml were resuspended in 1× PBS, 0.5% fetal calf serum. The EGFP-positive cells were sorted based on GFP intensity: low, mid and high. The cells were sorted into wells containing S2 conditioned medium prepared as described (Housden et al., 2015). The S2R+ EGFP-positive bulk sorted cells were amplified to populate 2× 10 cm dishes. To confirm the integration of the constructs, a fraction of the cells were harvested for genomic DNA isolation. PCR was performed using the following primers:

25C6/99F8 Primer 5, 5′-GAAGAACGGCATCAAGGTGAACTTCAAG-3′; 99F8 Primer 6, 5′-CGAAACGAATGGGAAATGGGATGGGATGC-3′; 99F8 Primer 7, 5′-ACGCTGCCAAATTGTTTGTCAGCTTCTCAC-3′; MTPIP82 to ACT5c FOR, 5′-ATTCGGTACCGAGCTCAAGCTTGCCGTTGCAGGACAGGATGTGGTGC-3′; MTPIP82 to ACT5c REV, 5′-CTGGCATAATTGTATGAGGCCTGCGTCGACACTAGTGGATCCAGACATG-3′.

### Generation of *bifocal* mutants

The design, creation and injection of the homology-directed repair construct was performed in conjunction with Rainbow Transgenic Flies Inc. Progeny of the injected individuals were screened for 3XP3-dsRED expression, and homologous recombination was confirmed by sequencing across the targeted junctions. The removal of the floxed cassette was confirmed by the loss of fluorescence and sequencing.

### Generation of anti-Bifocal antibody

The anti-Bifocal polyclonal antibody used in the study was custom generated by Genscript custom polyclonal antibody program, using amino acids 761-1107 of the Bifocal protein as the antigen. The antibodies were raised in two New Zealand strain rabbits, immunized with the *E. coli*-generated 761-1107 Bifocal peptide. Following three immunizations, antisera was collected and antigen affinity purified. The final concentration of the affinity purified antibody was 0.77 mg/ml.

### Immunofluorescence of tissue culture cells

*Drosophila* tissue culture cells were plated on poly-L-lysine-coated slides and allowed to attach for 4 h. They were fixed with 4% paraformaldehyde and washed with PBST-BSA buffer (0.1% Triton X-100 and 1% BSA) for 3×6 min incubations. Primary antibodies used [rabbit anti-PIP82 (Zelhof et al., 2020; 1:200), rabbit anti-Bifocal (1:200), mouse anti-HA (2367, Cell Signaling Technology; 1:200)] were added and incubated overnight at 4°C followed by 3×6 min washes with PBS-Triton X-100. Fluorescent-conjugated secondary antibodies obtained from Life Technologies (anti-mouse/rabbit Alexa Fluor 647 or Alexa Fluor 488; 1:200) were used and incubated for 2 h at room temperature followed by PBS-Triton X-100 washes. Confocal images were taken on a Leica SP8 or SP5 scanning confocal microscope.

### *Drosophila* husbandry and stocks

Stocks and crosses were maintained at 25°C. The following lines were used in this study: *w^1118^* [RRID: Bloomington *Drosophila* Stock Center (BDSC) 3605], Pph13-GAL4 (Liang et al., 2016), UAS-RNAi Pp1-87B (RRID: BDSC 32414) and UAS-Pp1-87B (RRID: BDSC 24098).

### Immunofluorescence staining of the pupal and adult retinas

48 h APF, 72 h APF and adult retinas were dissected and fixed in a fixative [PBS, 3.7% formaldehyde (pH 7.5)] for 20 min. After fixation, the pupal or adult retinas were washed 3× with PBS-T (PBS+0.1% Triton X-100) for 5 min each to remove the residual fixative. The retinas were then incubated 3× in block solution (PBS+0.1% Triton X-100+1% BSA) for 6 min each at room temperature. After blocking, the retinas were transferred into a 0.2 ml Eppendorf tube with 200 μl block solution containing primary antibodies and incubated overnight on a shaker at 4°C. After primary incubation, the retinas were washed 3× with block solution (6 min each). Secondary antibodies and phalloidin (1:200 dilution) were added in 200 μl block solution and incubated for 2 h on a shaker at room temperature. After secondary incubation, the tissues were washed once with PBS-T and then twice with PBS before mounting. The 48 h APF retinas were mounted between a cover slip and a glass slide; 72 h APF or later-staged retinas were mounted on a bridged glass slide to avoid crushing of the samples. Confocal images were taken on a Leica SP8 or SP5 scanning confocal microscope. Primary antibodies used were: anti-Eys (21A6 mouse monoclonal, AB 528449, Developmental Studies Hybridoma Bank; 1:50), anti-Crumbs [rat mouse monoclonal (Richard et al., 2006); 1:200], anti-Na^+^ K^+^ ATPase (a5 mouse monoclonal, AB 2166869, Developmental Studies Hybridoma Bank; 1:50) anti-PIP82 [rabbit anti-PIP82 (Zelhof et al., 2020); 1:100], anti-Armadillo (N2 7A1 mouse monoclonal, AB 528089, Developmental Studies Hybridoma Bank; 1:100), anti-Bifocal (rabbit anti-Bifocal, this study; 1:200) Fluorescent conjugated secondary antibodies were obtained from either Jackson ImmunoResearch or Life Technologies. Rhodamine or Alexa Fluor 647-conjugated phalloidin (Life Technologies; 1:200) was utilized for the detection of F-Actin.

### Western blotting

For western blot signal detection, horseradish peroxidase (HRP)-conjugated anti-mouse or anti-rabbit secondary antibody (Jackson ImmunoResearch; 1:5000) combined with Superscript West Pico Chemiluminescent Substrate (Thermo Fisher Scientific) was used. Primary antibodies used were as follows: rabbit anti-PIP82 (Zelhof et al., 2020; 1:1000), mouse monoclonal anti-alpha Tubulin (T9026, Sigma; 1:2500), rabbit anti-bifocal (antibody raised in rabbit against amino acids 761-1107; 1:1000), rabbit V5 tag (13202, Cell Signaling Technology; 1:1000) or mouse V5 tag (80076, Cell Signaling Technology; 1:1000). The original blots are shown in Fig. S9.

### TEM

Adult *Drosophila* heads were fixed in fix solution [4% paraformaldehyde, 3.5% glutaraldehyde, 2 mM CaCl_2_, 100 mM cacodylate buffer (pH 7.40)] with rocking overnight at 4°C. Heads were washed three times in 100 mM cacodylate buffer and post-fixed in 2% osmium tetroxide buffered with 100 mM cacodylate buffer for 1 h at room temperature. The heads were washed twice with 100 mM cacodylate buffer and once with dH_2_O, and then dehydrated through an ethanol series: once in 10%, 30%, 50%, 70%, and 90% ethanol, and then three times in 100% ethanol. The tissue was then incubated with propylene oxide for 2× 10 min followed by an incubation in 1:1 propylene oxide and Embed 812 resin (Electron Microscopy Sciences) overnight at room temperature. The tissue was incubated in Embed 812 resin for 8 h at room temperature, embedded in resin and incubated overnight at 65°C. The retina was sectioned with a Leica EM UC7 ultramicrotome and stained with 2% uranyl acetate in the dark for 20 min. After 3× 1 min washes in dH_2_O, the retina was stained with Reynold's lead citrate for 10 min in CO_2_-free chambers. The sample was washed once with CO_2_-free dH_2_O and then twice with dH_2_O, for 1 min each. After the sections were air dried, they were photographed with a JOEL 1400 plus transmission electron microscope.

### Sample (cells and heads) preparation for proteomics

For cell proteomics, experiments were performed in triplicates. S2R+ 99F8 MT PIP82-TID cells were induced with 500 μM copper sulphate for 72 h for the expression of PIP82-TID. 100×10^6^ cells were utilized, and the cell pellet was lysed by resuspending in 500 μl RIPA lysis buffer [50 mM Tris-HCl pH 7.5, 300 mM NaCl, 0.1% SDS, 0.5% sodium deoxycholate, 1% NP-40, 1 mM EDTA, 1 mM DTT, 1× protease inhibitor cocktail (Thermo Fisher Scientific)]. It was then incubated for 10 min at 4°C with the buffer, after which sonication was carried out (five duty cycles). Lysates were clarified by centrifugation at 12,000 ***g*** for 10 min at 4°C. Supernatant was further used for SDS gel, western blot and enrichment experiments. For *Drosophila* heads, experiments were performed in triplicates. One day post-eclosion, 100 adult flies were collected in a 15 ml Falcon tube. The tube was immersed in liquid nitrogen to freeze the flies. The tube was then vortexed to fragment the fly bodies from heads. The heads were microscopically separated and collected in an Eppendorf tube with 200 μl RIPA lysis buffer containing protease inhibitors on ice. The samples were macerated thoroughly with a sterilized pestle followed by sonication (five duty cycles) to lyse. The lysates were incubated for 10 min at 4°C and clarified by centrifugation at 14,000 rpm for 10 min at 4°C. The supernatants were used for SDS gel, western blot and enrichment experiments.

### Streptavidin bead enrichment of biotinylated material

For enrichment of biotinylated material, 75 μl streptavidin-coated magnetic beads (Dynabeads™ MyOne™ Streptavidin C1, Invitrogen) were washed twice with RIPA buffer, then incubated with clarified lysates containing ∼1 mg protein for each sample with rotation overnight at 4°C. The beads were subsequently washed twice with 1 ml RIPA lysis buffer, once with 1 ml of 1 M KCl, once with 1 ml of 0.1 M Na_2_CO_3_, once with 1 ml of 2 M urea in 10 mM Tris-HCl (pH 8.0), and twice with 1 ml RIPA lysis buffer. For quality control analysis, biotinylated proteins were eluted by boiling 5% of the beads in 50 μl of 2× protein-loading buffer supplemented with 20 mM DTT and 2 mM biotin and run on SDS-PAGE gel. Western blot analysis was performed by probing with streptavidin HRP (Invitrogen) and detection by the chemiluminescent method (Pierce™ ECL Western Blotting Substrate). For identification of bound proteins by proteomics, after RIPA lysis buffer washes, beads were washed with 10 mM Tris-HCl pH 7.5 (2×2 min), supernatant was removed, and the beads were submitted to the Laboratory for Biological Mass Spectrometry facility at IU Bloomington.

### Mass spectrometry

Individual immunoprecipitated pellets were denatured in 8 M urea in 100 mM ammonium bicarbonate. Samples were incubated for 45 min at 57°C with 10 mM Tris(2-carboxyethyl) phosphine hydrochloride to reduce cysteine residue side chains. These side chains were then alkylated with 20 mM iodoacetamide for 1 h in the dark at 21°C. The urea was diluted to 1 M urea using 100 mM ammonium bicarbonate. A total of 0.4 µg trypsin (Promega) was added, and the samples were digested for 14 h at 37°C. The resulting peptide solution was desalted using ZipTip pipette tips (EMD Millipore), dried down and resuspended in 0.1% formic acid. Peptides were analyzed by LC-MS on an Orbitrap Fusion Lumos equipped with an Easy NanoLC1200. Buffer A was 0.1% formic acid in water. Buffer B was 0.1% formic acid in 80% acetonitrile. Peptides were separated on a 30 min gradient from 0% B to 35% B. Peptides were fragmented by higher-energy collision dissociation (HCD) at a relative collision energy of 32%. Precursor ions were measured in the Orbitrap with a resolution of 60,000. Fragment ions were measured in the Orbitrap with a resolution of 15,000. Data were analyzed using Proteome Discoverer (2.5) to interpret and quantify the relative amounts in a label-free quantification manner. Data were searched against the *Drosophila melanogaster* UniProt proteome downloaded in July 2020. Trypsin was set as the protease with up to two missed cleavages allowed. Carbamidomethylation of cysteine residues was set as a fixed modification. Oxidation of methionine and protein N-terminal acetylation were set as variable modifications. A precursor mass tolerance of 10 ppm and a fragment ion quantification tolerance of 0.04 Da were used. Data were quantified using the Minora feature detector node within Proteome Discoverer. All raw data can be found in Tables S3 and S4.

Data were filtered to remove protein identifications with less than three peptides (cells) or two peptides (heads), and Q-values greater than 0.001. SAINTexpress (Teo et al., 2014) was run using default parameters on the CRAPome database website (Mellacheruvu et al., 2013). Network data were downloaded from the CRAPome output, and the interaction network was generated using Cytoscape 3 (Shannon et al., 2003). Gene Ontology enrichment analysis was performed using PANGEA with default parameters.

## Supplementary Material

10.1242/joces.262223_sup1 Supplementary information

Table S3. Mass spectrometry Raw Data

Table S4. Mass spectrometry Raw Data
