# Supplementary material for: Proximity labeling reveals interactions necessary to maintain the distinct apical domains of Drosophila photoreceptors
Source: J Cell Sci. 2024 Dec 11;137(23):jcs262223. doi: 10.1242/jcs.262223 (PMC11827603; doi:10.1242/jcs.262223)
Supplement: Supplementary information [file joces-137-262223-s1.pdf]

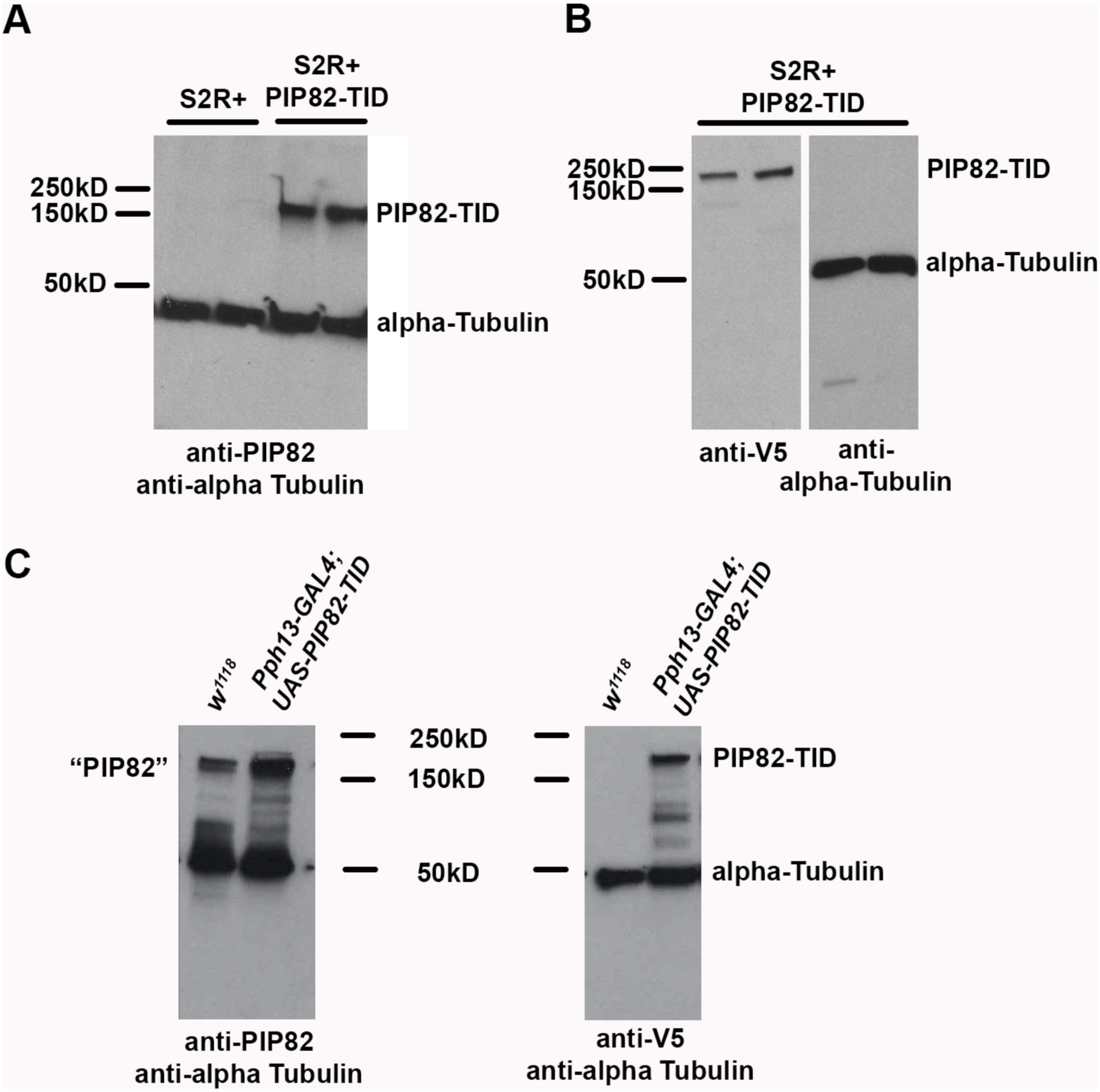

Journal of Cell Science • Supplementary information

Fig. S1. Western analysis of PIP82-TurboID expression in *Drosophila* tissue culture cells and photoreceptor cells. A. Western blot analysis of S2 extract from control or stable cell line containing the PIP82-TID construct in the presence of Cu^{++}. Antibodies against PIP82 and alpha-Tubulin were utilized. **B.** Western blot analysis of S2 extract from the stable cell line containing the PIP82-TID construct in the presence of Cu^{++}. Antibodies against the V5 epitope and alpha-Tubulin were utilized. **C.** Western blot analysis of *Drosophila* head extract from wild type (w^{1118}) or transgenic line expressing (*Pph13-GAL4/+;UAS-PIP82-TID*/+) PIP82-TID in photoreceptors. Antibodies against PIP82 or the V5 epitope were used to identify endogenous PIP82 and PIP82-TID proteins and antibodies against alpha-Tubulin were utilized for loading controls. Native PIP82 is 132kDa and the addition of the V5 epitope tag and TurboID adds an additional 35kDa.

Journal of Cell Science • Supplementary information

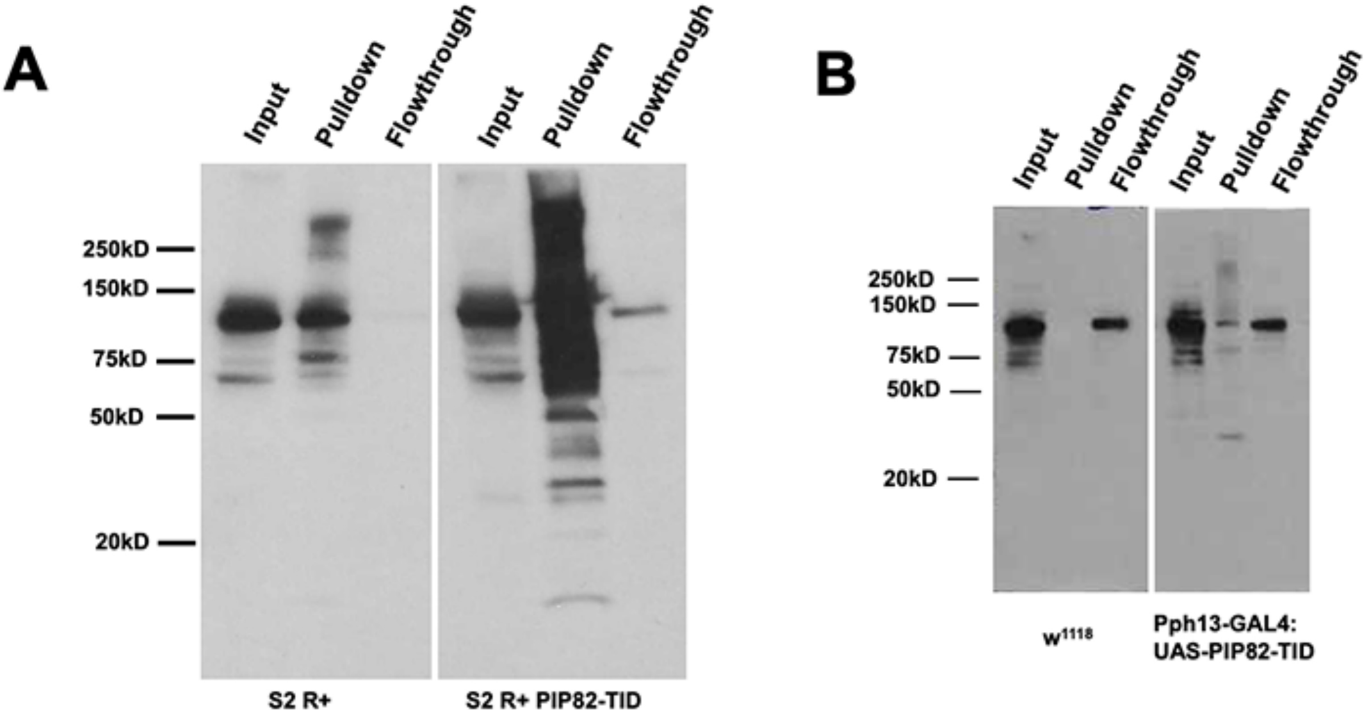

Fig. S2. Western analysis of enrichment of biotinylated proteins associated with the expression of PIP82-TurboID. A. Western blot analysis of biotinylated proteins from S2 R+ cells with or without PIP82-TurboID. **B.** Western blot analysis of biotinylated proteins from photoreceptor cells with or without PIP82-TurboID.

Journal of Cell Science • Supplementary information

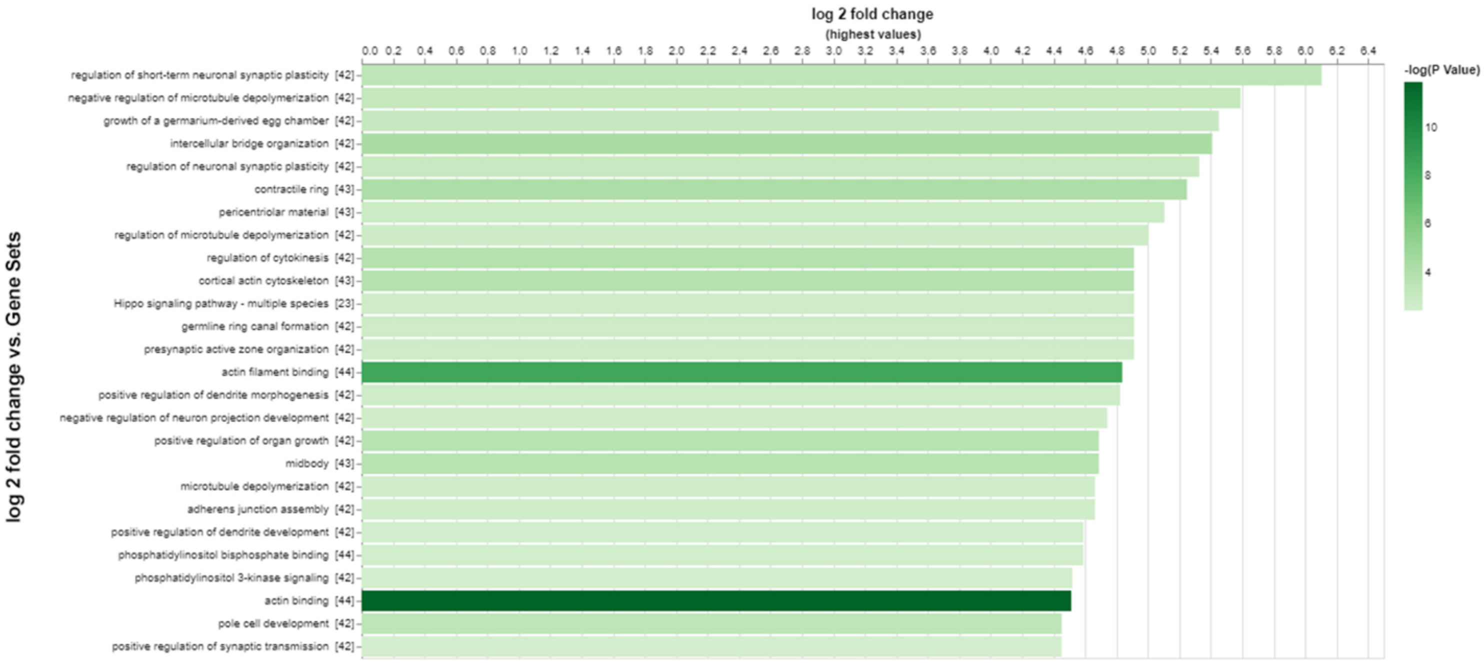

Fig. S3. PAthway, Network and Gene-set Enrichment Analysis (PANGEA) of PIP82 interactome.

Journal of Cell Science • Supplementary information

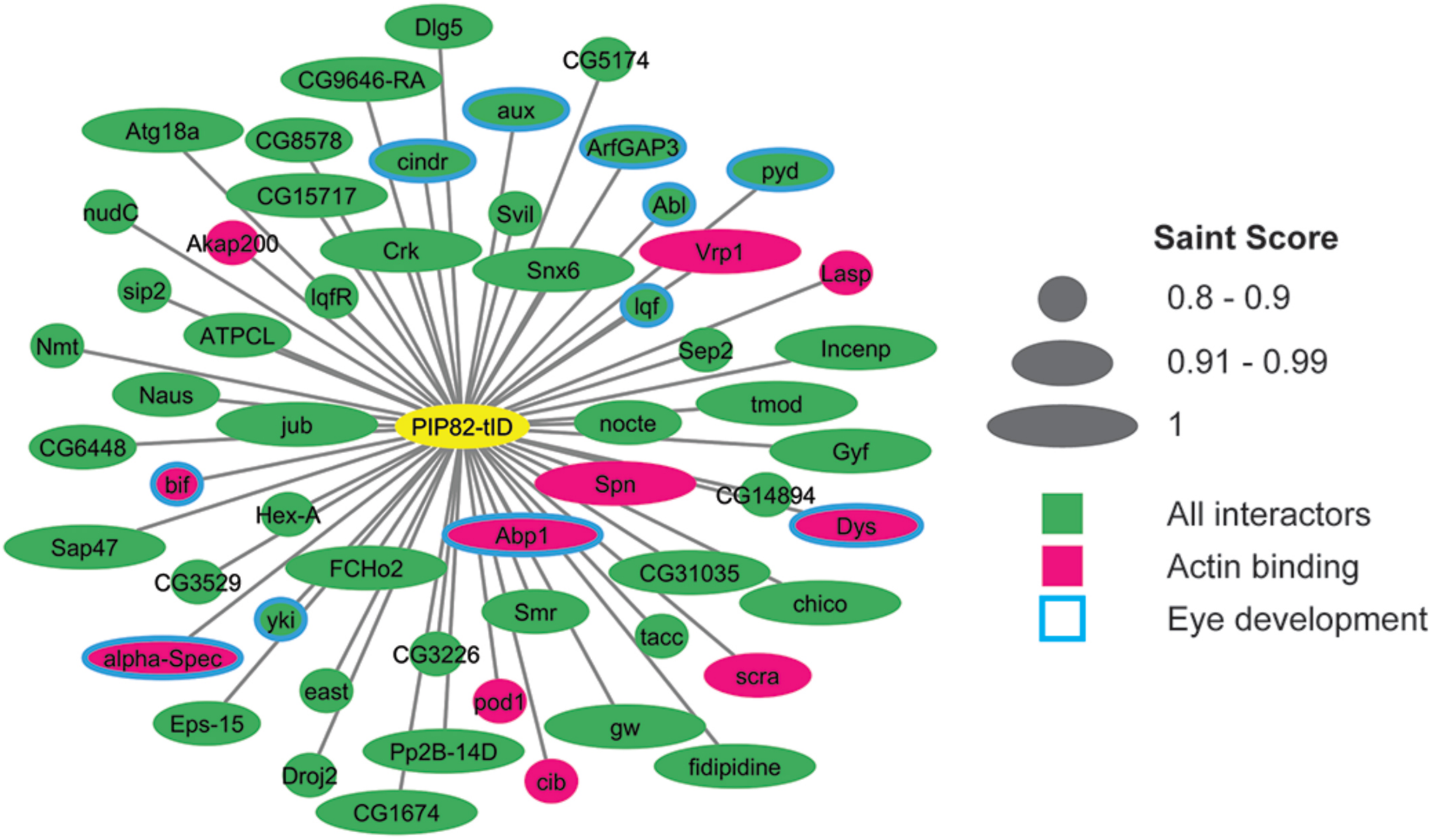

Fig. S4. PIP82 S2 cell interactome. Representation of the 58 PIP82 interactors and associated SAINT score. The interactors associated with Actin are shaded in magenta and those implicated in eye development are outlined in blue.

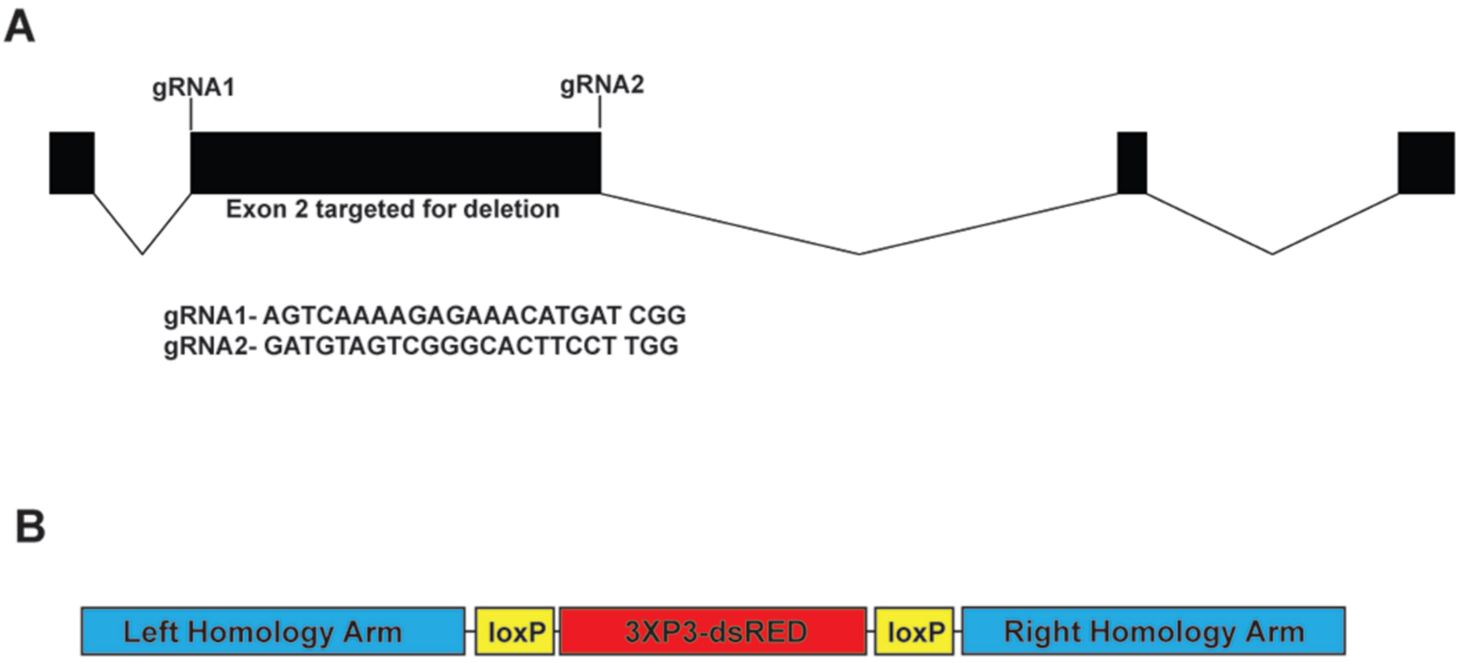

C

MESQKRPSLDLHTDVPAGLAAGGSGLGAAAEMSPTSGFLPDMPQWKKDLIQRRKTNVA
RTQAASITSPTDGSCGALAEANAAPGAIADFTEPATISSTSQKRNMGS*ARLHRKRRCDLQ*
*AQAGAAKAK**

Fig. S5. Design schematic for generating a CRISPR/Cas9 null allele for *bifocal*. A. Scaled exon/intron schematic of *bifocal* gene. gRNAs were designed to flank the second exon. **B.** Design of the homologous recombination repair construct. Each arm is approximately 1kb and is designed to replace the entire second exon with a floxed cassette containing the 3XP3 promoter driving dsRED, detected by fluorescence in photoreceptors. **C.** Resulting Bifocal protein sequence upon removal of the second exon. The non-italic amino acids are endogenous Bifocal amino acids, and italic amino acids represent exogenous amino acids due to the frameshift created by the deletion.

Journal of Cell Science • Supplementary information

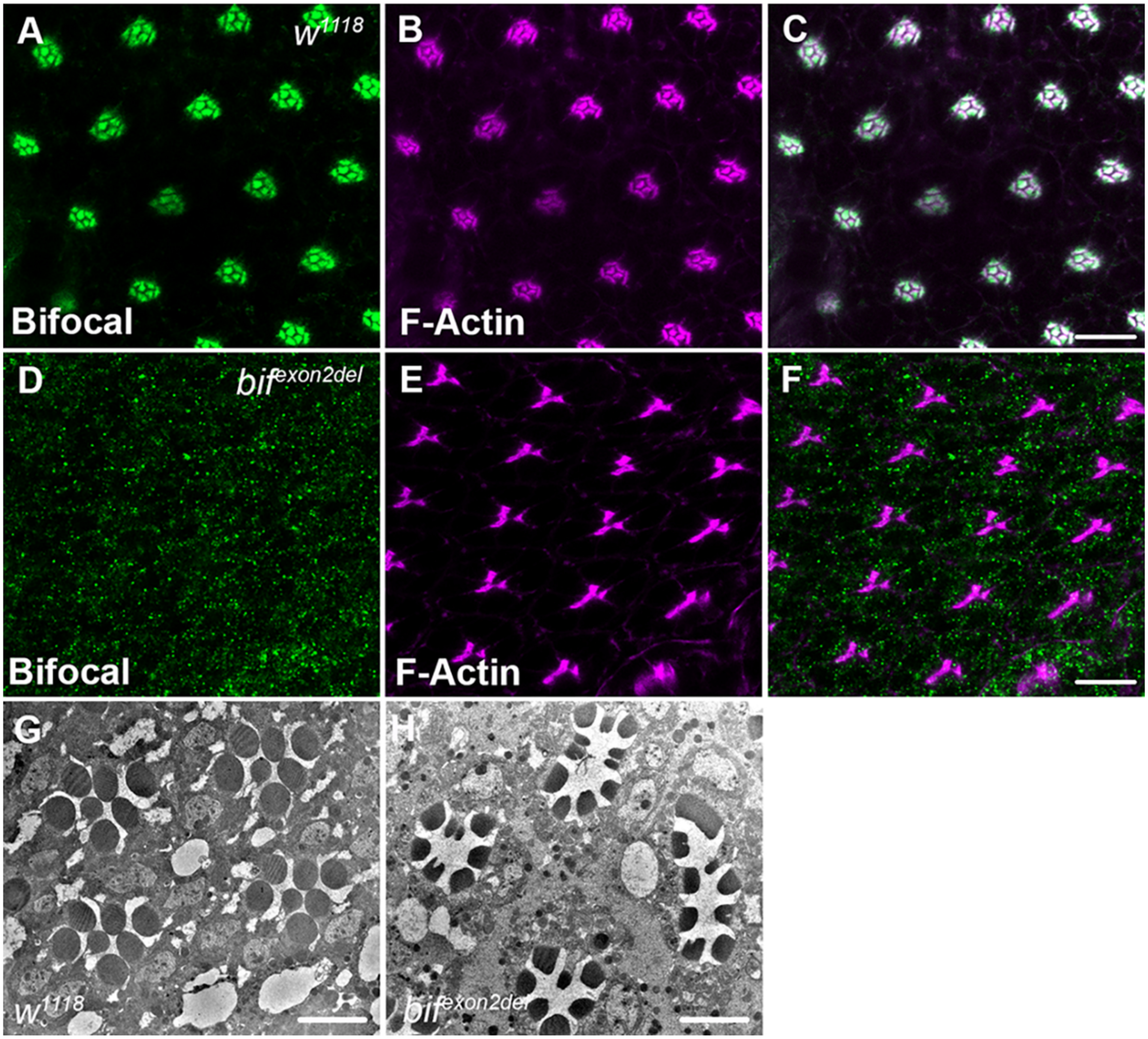

Fig. S6. Confirmation and phenotypic analysis of *bif*^{exon2del} mutant and Bifocal antibody specificity. **A-C.** Immunostaining of Bifocal (green) and F-Actin (magenta) in a wild-type retina at 48 hrs after puparium formation. **D-F.** Immunostaining of Bifocal (green) and F-Actin (magenta) in a *bif*^{exon2del} mutant retina at 48 hrs after puparium formation. Scale bars 10um. **G.** Transmission electron micrograph of a wild-type adult retina. **H.** Transmission electron micrograph of a *bif*^{exon2del} mutant retina. Scale bars 5 um.

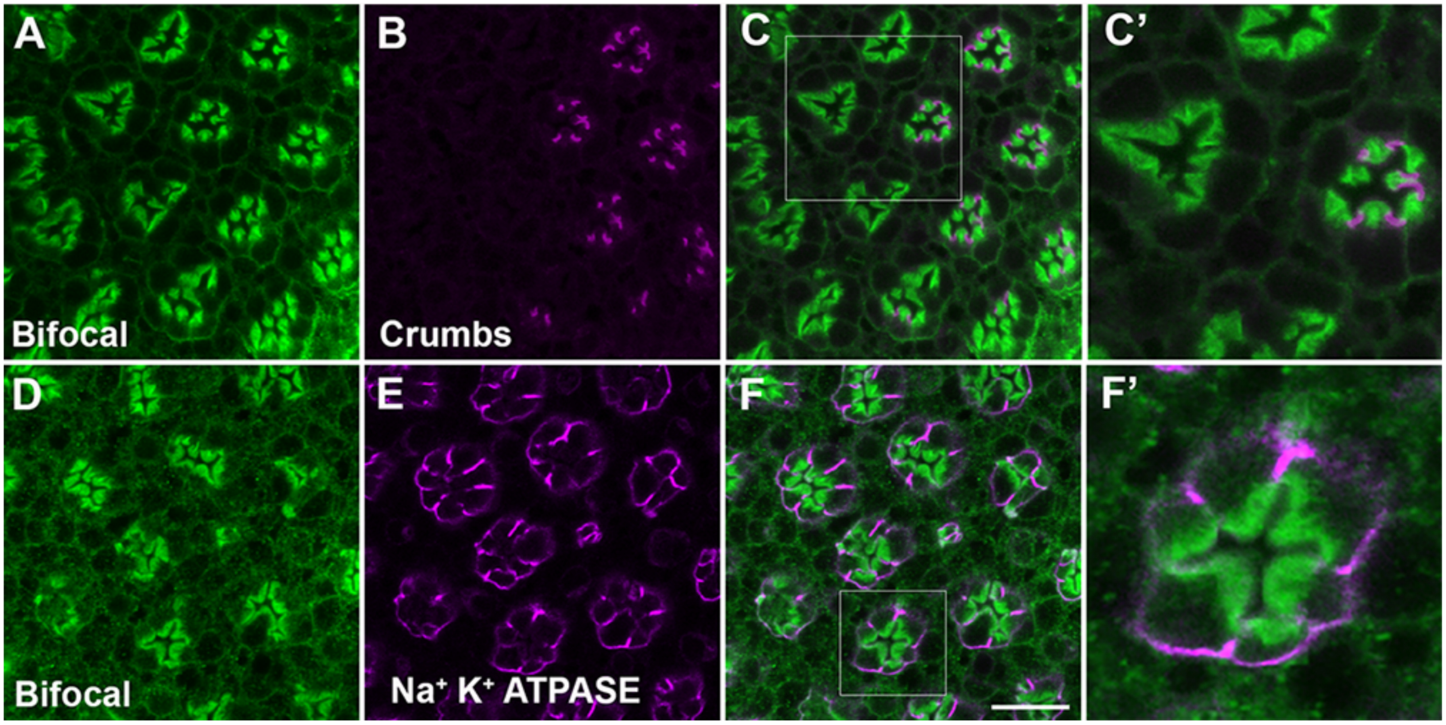

Fig. S7. Crumbs is required for proper restriction of Bifocal on the apical membrane. A-C. Immunostaining of Bifocal (green) and Crumbs (magenta) in mosaic clones containing both wildtype and mutant *crumb* photoreceptors at 84 hrs APF. Note in the merged image **C,C'** upon the loss of Crumbs, Bifocal localization now extends the entire apical membrane. Analysis of Bifocal localization in *crumbs* mutant photoreceptor cells. **D-F.** Immunostaining of Bifocal (green) and alpha subunit Na^+ K^+ ATPase (magenta) in mosaic clones containing both wildtype and mutant *crumb* photoreceptors at 84 hrs APF. The loss of Crumbs does not change the localization pattern of alpha subunit Na^+ K^+ ATPase. Scale Bar 10um**.**

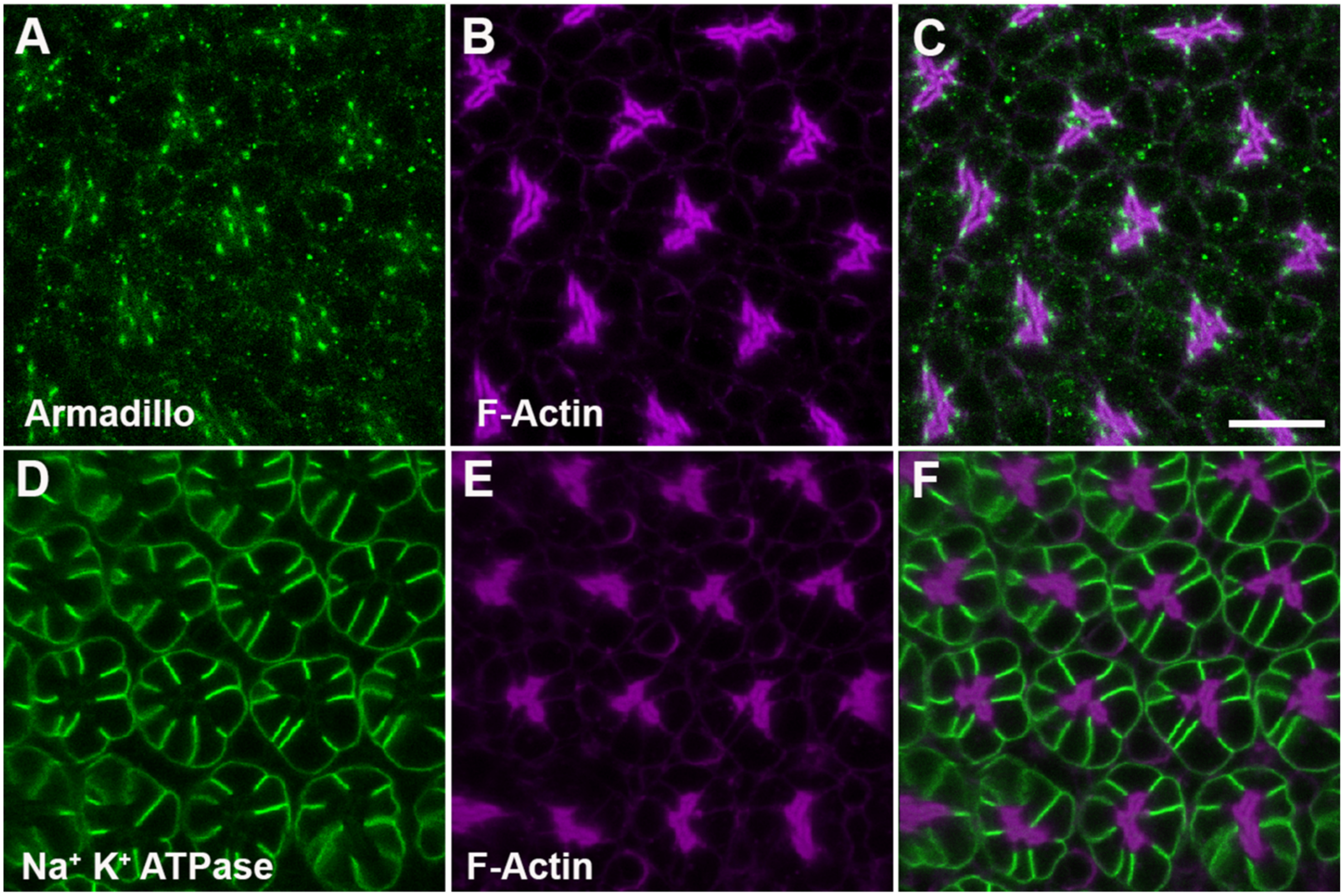

Fig. S8. Adherence junction formation and trafficking to the basal-lateral membrane is normal in *bifocal;PIP82* double mutant photoreceptors. A-C. Immunostaining of Armadillo (green) and F-Actin (magenta) in *bifocal;PIP82* double mutant photoreceptors at 72hrs APF. **D-F.** Immunostaining of the alpha subunit Na^+ K^+ ATPase (green) and F-Actin (magenta) in *bifocal;PIP82* double mutant photoreceptors at 72hrs APF. Scale bar 10 um.

Supp Figure 1A

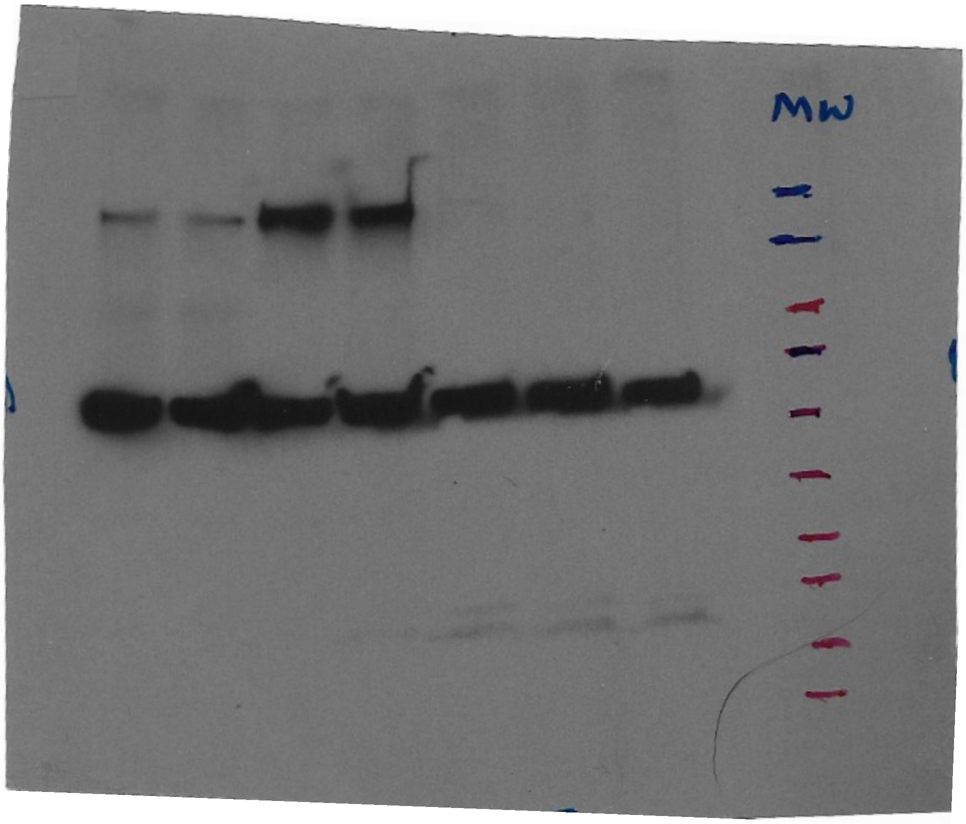

Journal of Cell Science • Supplementary information

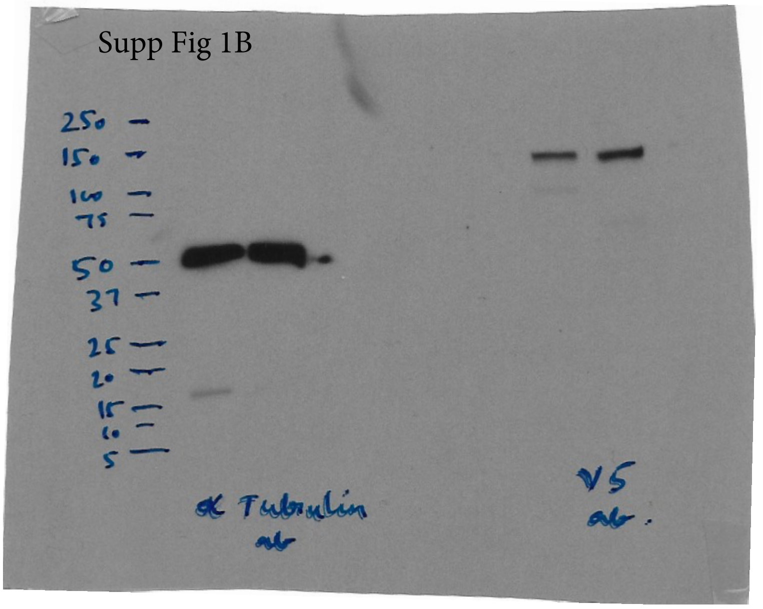

Supp Fig 1C

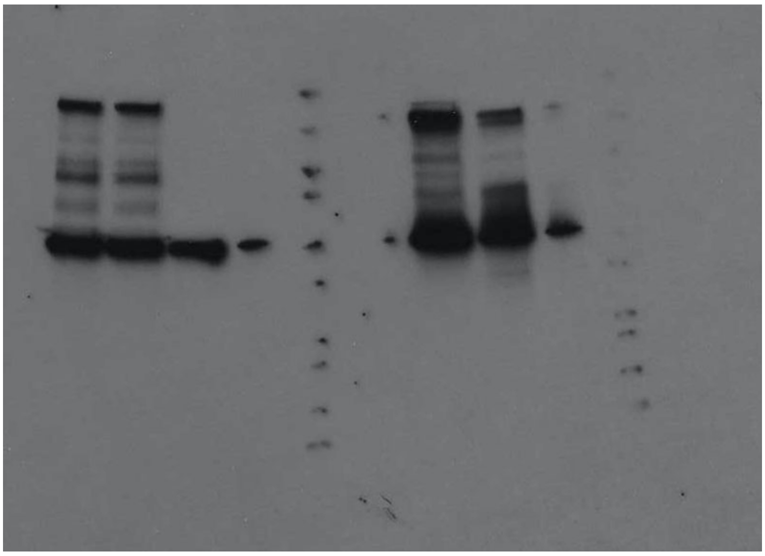

Journal of Cell Science • Supplementary information

Supp Fig 2A

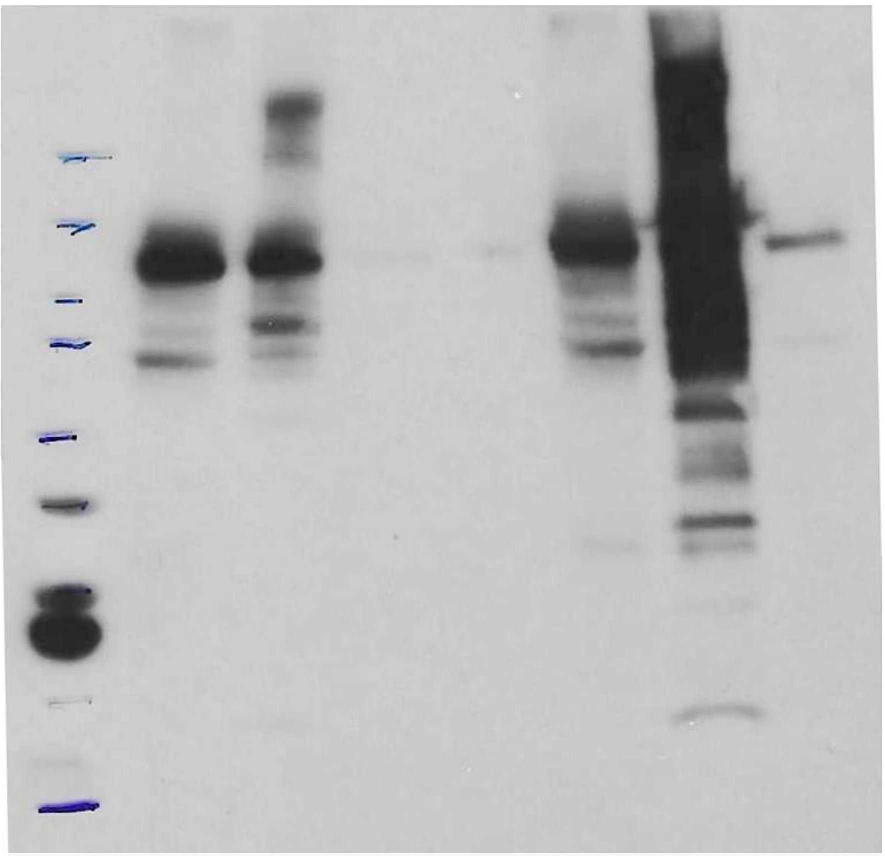

Supp Fig 2B

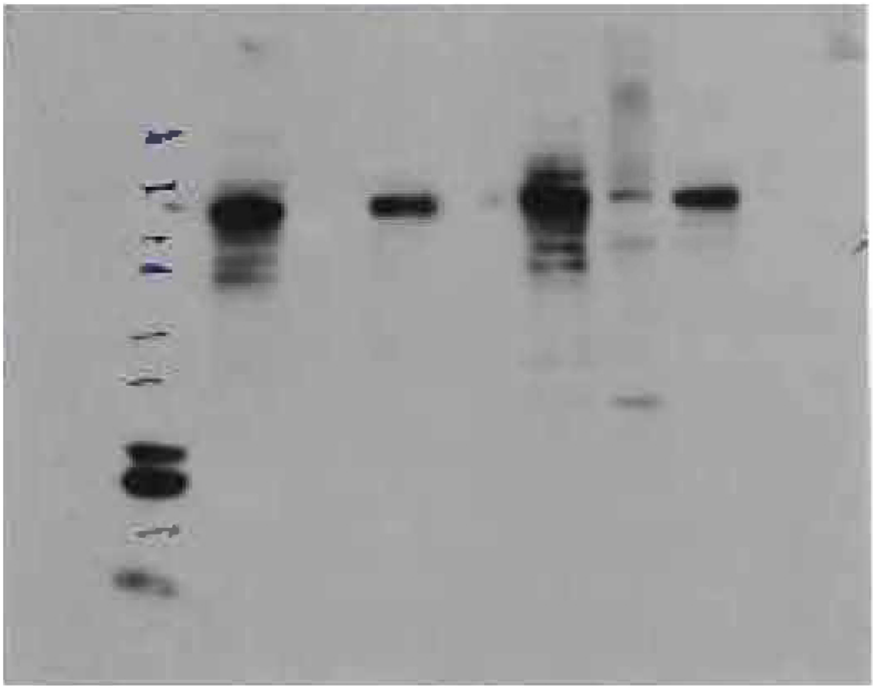

Fig. S9. Blot transparency

Table S1. PIP82 photoreceptor protein interactome.

The table lists the proteins which were enriched upon expression of PIP82-TurboID in adult fly retinas versus the w^{1118} control either exclusively or over at least 3-fold. ND=not detectable, NA=not applicable. Listed for each protein in the table are the average values from the four replicates. Coverage- the percentage of the protein that is covered by the identified peptides. Sum PEP score - calculated based on the posterior error probability (PEP) values of the peptide spectrum matches. Number of peptides and Abundance values. The Abundance values are a semi-quantitative measure of the amount of each specific protein in a given sample. The fold difference represents the average fold difference between each specific protein between the control (w1118) and experimental. Only those that showed a ≥ 3-fold difference are listed.

						Abundance values		
**Accession**	**Description**	**Gene**	**Sum PEP Score**	**Coverage [%]**	**# Peptides**	**W1118 Control**	**PIP82 - tid**	**Fold difference**
M9PHA0	Bifocal, isoform F	bif	10.63	3.75	3	ND	1.88E+07	NA
Q9NB04	Patj homolog	Patj	10.84	4.75	3	8.64E+04	5.06E+07	586
X2JC02	Supervillin, isoform AB	Svil	6.97	0.75	2	5.87E+04	2.08E+07	354
A1Z8H7	Cuticular protein 47Ef, isoform C	Cpr47Ef	28.51	15.25	3	8.04E+06	6.17E+07	8
Q9VMV5	Viking, isoform A	vkg	11.63	2.75	4	1.09E+07	7.45E+07	7
A0A0B4LFX4	Coracle, isoform F	cora	23.05	5.50	7	2.44E+07	1.05E+08	4
Q9VSN3	Cuticular protein 66D	Cpr66D	16.72	20.50	4	4.85E+07	1.75E+08	4
Q7KVX5	VAMP-associated protein 33kDa, isoform A	Vap33	10.31	13.00	4	2.96E+07	9.17E+07	3
Q9VV46	Cuticular protein 72Ec	Cpr72Ec	69.38	37.50	18	8.54E+08	2.59E+09	3
Q9VV36	Retinin	retinin	29.08	30.75	5	5.48E+08	1.64E+09	3
Q9V7U0	Pro-resilin	resilin	8.75	4.75	2	4.03E+07	1.14E+08	3
Q9VZG0	Cuticular protein 64Ac	Cpr64Ac	15.03	26.25	3	8.29E+07	2.23E+08	3
P19889	60S acidic ribosomal protein P0	RpLP0	4.21	5.75	2	2.09E+06	5.46E+06	3

Journal of Cell Science • Supplementary information

Table S2. PIP82 S2 Cell Interactome

Protein ID	Gene	FC-A	FC-B	SAINT P
Q9VYG8	CG15717	81.3	59.1	1
A1ZAC7	fidipidine	36.8	36.6	1
M9PER1	Atg18a	125.3	124.9	1
Q7KQM6	Gyf	53.9	34.8	1
Q9VHC4	FCHo2	250.0	164.0	1
Q1RKY9	CG1674	69.7	52.8	1
M9MSA2	jub	52.7	47.2	1
A0A0B4KGH0	Dys	28.8	19.6	1
A1ZAP1	CG9646-RA	108.1	93.4	1
Q7JRJ9	Incenp	117.7	95.0	1
A0A0B4K6N4	tmod	88.6	74.9	1
M9PCQ8	chico	112.8	64.3	1
Q8SY33	gw	116.5	78.7	1
M9NE01	Pp2B-14D	65.3	52.7	1
Q7KVL6	Vrp1	110.2	97.6	1
M9PGV6	alpha-Spec	151.7	145.0	1
Q9XYM0	Crk	268.1	266.5	1
M9NFI2	Spn	10.4	10.3	1
Q8IMK1	CG31035	68.2	47.6	1
Q9VLQ9	Snx6	77.8	77.0	1
Q9VU84	Abp1	123.7	108.6	1
Q960T2	Sap47	50.6	49.9	1
Q9W3E2	PIP82	30.0	29.1	1
Q8SX68	Naus	1078.7	982.2	0.98
Q9VHK1	pyd	112.7	110.3	0.98
Q9VXN3	CG8578	173.1	172.9	0.97
A0A0B4KG14	aux	33.1	24.1	0.96
M9PCQ6	Dlg5	148.4	114.1	0.96
Q9VYK0	Smr	51.9	20.3	0.96
M9PGG0	ArfGAP3	308.1	215.8	0.96
Q9W2U7	nocte	513.2	487.4	0.95
Q7KN85	ATPCL	111.7	109.8	0.94
Q9V4P1	scra	247.2	239.9	0.94
R9PY70	CG6448	30.8	30.0	0.93
Q8MMD3	Eps-15	276.2	275.5	0.92
Q9VA36	cindr	276.0	236.2	0.91
Q9W016	Svil	380.0	151.6	0.89
Q9VFV9	Droj2	44.7	41.1	0.89
M9PEM7	lqf	193.3	168.8	0.88
Q9VD13	lqfR	227.7	203.5	0.88
A0A0C4DHD4	yki	117.1	97.0	0.87
P54359	Sep2	67.7	58.7	0.86
A8JV09	pod1	87.9	73.7	0.86
Q9VEV3	CG14894	388.3	362.6	0.85
O61613	Nmt	186.6	185.9	0.84
Q9VSZ1	CG3529	116.7	105.2	0.83
Q86BM5	Akap200	97.0	92.3	0.83
Q8I7C3	Lasp	1462.9	1427.6	0.82
O46048	east	79.2	72.9	0.82
O97428	cib	80.9	80.7	0.82
A0A0B4JD97	tacc	113.8	112.4	0.81
Q9W3Y3	CG3226	140.8	115.4	0.81
Q9VVA6	nudC	81.6	80.1	0.81
M9PCT8	Abl	149.6	142.5	0.81
Q9W330	Hex-A	65.1	62.0	0.81
Q7JRI6	CG5174	5.1	4.3	0.8
Q8IR86	bif	47.4	46.0	0.8
Q7KTL5	sip2	498.0	496.1	0.8

The table lists the proteins which were enriched upon expression of PIP82-TurboID in S2 cells based upon SAINTexpress analysis utilizing default parameters on the CRAPome website. Proteins that had a SAINT P > .8 are listed. FC-A and FC-B were calculated using the CRAPome Empirical Fold Change Score tool.

Journal of Cell Science • Supplementary information

Table S3. Mass spectrometry Raw Data

Available for download at
https://journals.biologists.com/jcs/article-lookup/doi/10.1242/jcs.262223#supplementary-data

Table S4. Mass spectrometry Raw Data

Available for download at

https://journals.biologists.com/jcs/article-lookup/doi/10.1242/jcs.262223#supplementary-data

Journal of Cell Science • Supplementary information
